# Dynamic alterations in decoy VEGF receptor-1 stability regulate angiogenesis

**DOI:** 10.1038/ncomms15699

**Published:** 2017-06-07

**Authors:** Joshua M. Boucher, Ryan P. Clark, Diana C. Chong, Kathryn M. Citrin, Lyndsay A. Wylie, Victoria L. Bautch

**Affiliations:** 1 Department of Biology, University of North Carolina at Chapel Hill, Chapel Hill, North Carolina 27599, USA; 2 Lineberger Comprehensive Cancer Center, University of North Carolina at Chapel Hill, Chapel Hill, North Carolina 27599, USA; 3 Genetics and Molecular Biology Curriculum, University of North Carolina at Chapel Hill, Chapel Hill, North Carolina 27599, USA; 4 McAllister Heart Institute, University of North Carolina at Chapel Hill, Chapel Hill, North Carolina 27599, USA

## Abstract

Blood vessel expansion is driven by sprouting angiogenesis of endothelial cells, and is essential for development, wound healing and disease. Membrane-localized vascular endothelial growth factor receptor-1 (mVEGFR1) is an endothelial cell-intrinsic decoy receptor that negatively modulates blood vessel morphogenesis. Here we show that dynamic regulation of mVEGFR1 stability and turnover in blood vessels impacts angiogenesis. mVEGFR1 is highly stable and constitutively internalizes from the plasma membrane. Post-translational palmitoylation of mVEGFR1 is a binary stabilization switch, and ligand engagement leads to depalmitoylation and lysosomal degradation. Trafficking of palmitoylation enzymes via Rab27a regulates mVEGFR1 stability, as reduced levels of Rab27a impaired palmitoylation of mVEGFR1, decreased its stability, and elevated blood vessel sprouting and *in vivo* angiogenesis. These findings identify a regulatory axis affecting blood vessel morphogenesis that highlights exquisite post-translational regulation of mVEGFR1 in its role as a molecular rheostat.

## Abstract (web summary)

Membrane-bound mVEGFR1 is a decoy VEGF-A receptor that regulates VEGF-A signalling amplitude. Boucher *et al*. show that Rab27a-regulated palmitoylation of mVEGFR1 redirects the receptor from a stable, constitutively recycling mode to a degradative route that removes ligands from the system.

New blood vessel formation from existing vessels via endothelial cell sprouting is called angiogenesis, and enables vessels to dynamically respond to tissue requirements for oxygen and nutrients^1^,^2^,^3^. Vascular Endothelial Growth Factor-A (VEGF-A)-mediated signalling is crucial for angiogenesis^4^,^5^. Local production of VEGF-A stimulates angiogenesis upon binding to endothelial cell-expressed vascular endothelial growth factor receptor-2 (VEGFR2)^6^. Tight control of VEGF-A signalling is required for proper vessel growth and patterning, and increased VEGF-A signalling is linked to disease^7^,^8^.

A second vascular endothelial growth factor receptor, VEGFR1, is alternatively spliced to yield both soluble (sVEGFR1) and membrane-tethered (mVEGFR1) isoforms^9^. Both isoforms bind VEGF-A with tenfold higher affinity than VEGFR2, but mVEGFR1 has weak kinase activity in endothelial cells and signalling appears confined to pathological situations^10^,^11^,^12^,^13^,^14^. Signalling through mVEGFR1 is also important in bone-marrow-derived cells such as macrophages for homing to sites of inflammation and ischaemia^15^,^16^. Two other ligands, VEGF-B and platelet-derived growth factor (PLGF), bind exclusively to VEGFR1; VEGF-B binding does not lead to detectable signalling, whereas PLGF binding can produce a signal^17^,^18^. Mice genetically lacking VEGFR1 (*Flt-1* in the mouse) die prenatally from overgrown blood vessels^19^, but mice lacking the signalling domain of *mFlt-1* are viable and fertile^20^, indicating that mVEGFR1 signalling is not required for vascular development. Genetic rescue of either *mFlt-1* or *sFlt-1* reduces VEGFR2 signalling and mitigates the overgrowth of embryonic stem cell-derived *Flt-1*^*−/−*^ mutant vessels^21^, supporting that both VEGFR1 isoforms function as molecular rheostats to negatively modulate VEGFR2 signalling during developmental angiogenesis.

Although the consequences of VEGFR1 loss are well-documented, how mVEGFR1 availability is controlled to modulate signalling is unknown. Post-translational modifications diversify protein function and specify localization and turnover^22^. Post-translational regulation of VEGFR2 is well-characterized; for example, phosphorylation and ubiquitination are important for signalling^23^,^24^,^25^. However, little is known about how mVEGFR1 is regulated post-translationally. It was reported that mVEGFR1 is largely Golgi-resident in cultured cells, that it does not recycle, and that ligand-bound mVEGFR1 is ubiquitinated and degraded^26^,^27^,^28^. It is unknown whether VEGFR1 post-translational modifications regulate VEGF signalling amplitude. One lipid post-translational modification, palmitic acid addition to cysteines, or palmitoylation, is unique in that it is reversible, thereby enabling dynamic regulation of protein stability^29^. eNOS palmitoylation influences vascular homeostasis^30^, and PECAM-1 palmitoylation promotes cell surface localization in endothelial cells^31^. In other cell types, palmitoylation prevents Fas receptor degradation^32^, regulates ligand binding and signalling of EGFR^33^,^34^, and controls intracellular trafficking and stability of c-MET^35^.

Here we show that mVEGFR1 is unusually stable and undergoes constitutive internalization in endothelial cells. Palmitoylation acts as a binary switch to regulate mVEGFR1 stability and trafficking via the palmitoylating enzyme DHHC3 and the depalmitoylating enzyme APT1. We identify the small GTPase Rab27a as an upstream regulator of mVEGFR1 palmitoylation. Rab27a loss impairs mVEGFR1 palmitoylation, increases mVEGFR1 trafficking to lysosomes for degradation and perturbs vascular morphogenesis *in vitro* and *in vivo*. These findings identify a unique angiogenic regulatory axis centred on post-translational regulation of mVEGFR1 that is consistent with its role as a molecular rheostat for VEGF-A signalling, and show that post-translational regulation of a decoy receptor affects biological outputs.

## Results

### mVEGFR1 is stable and constitutively cycles

**Figure 1:**
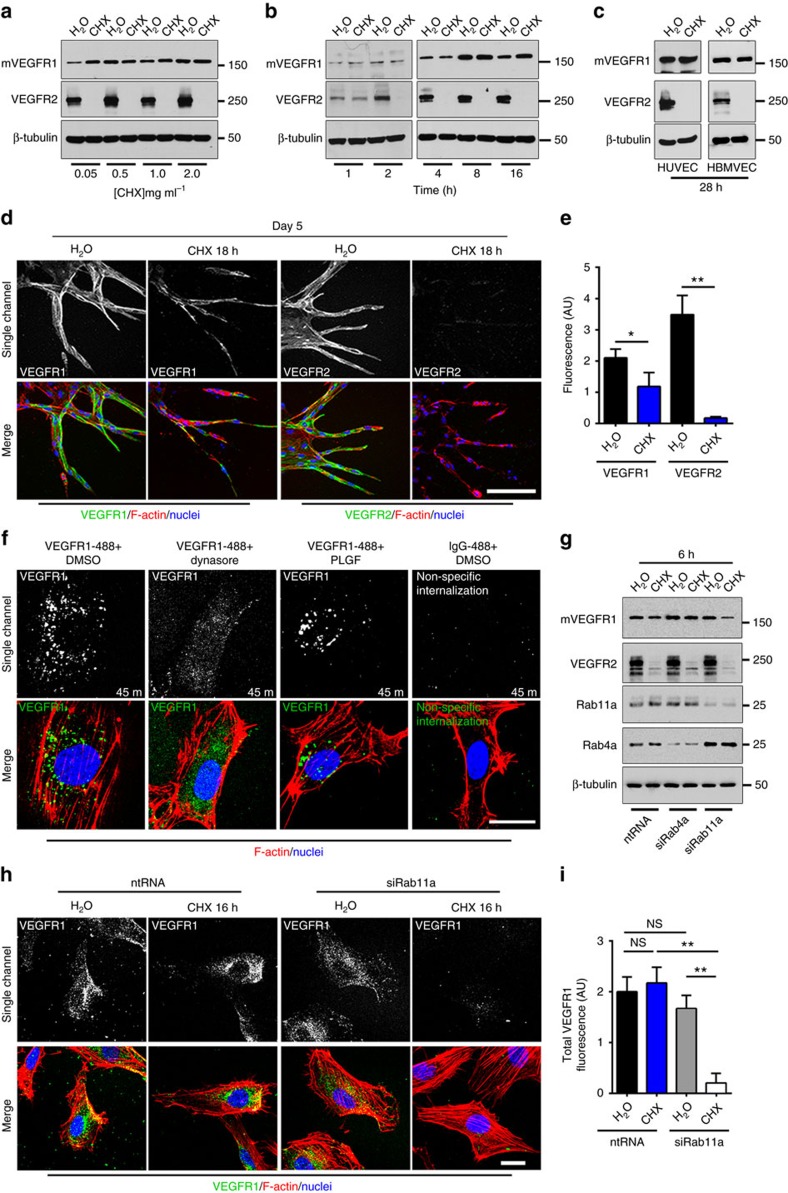
mVEGFR1 is stable and constitutively internalizes in endothelial cells. (**a**) Immunoblot of HUVEC treated as indicated for 8 h; CHX, cycloheximide; three replicates. (**b**) Immunoblot of HUVEC treated as indicated; three replicates. (**c**) Immunoblot of HUVEC or HBMVEC with indicated treatments; two replicates. (**d**,**e**) (**d**) Immunofluorescence for VEGFR1 or VEGFR2 in HUVEC angiogenic sprouts with indicated treatments, scale bar: 100 μm. (**e**) Quantification of fluorescence via integrated density. (VEGFR1 *n*=18 sprouts per condition; VEGFR2, *n*=25 sprouts per condition); three replicates. (**f**) VEGFR1 immunofluorescence of HUVEC with indicated treatments; three replicates, scale bar: 20 μm. (**g**) Immunoblot of HUVEC at 48 h post-knockdown (KD) with indicated treatments; two replicates. (**h**,**i**) (**h**) VEGFR1 immunofluorescence of HUVEC 24 h post KD with indicated treatments, scale bar: 20 μm. (**i**) Quantification of fluorescence via integrated density. (No. of cells: ntRNA/H_2_O, *n*=22; ntRNA/CHX, *n*=23; siRab11a/H_2_O, *n*=19; siRab11a/CHX, *n*=14); three replicates. Statistics: Shown are means +95% CI. One-way ANOVA with pairwise comparison and post-hoc Tukey's range test. *P≤0.05; **P≤0.01; NS, not significant.

Because mVEGFR1 and VEGFR2 have distinct functions in VEGF-A signalling, we hypothesized that they have differential post-translational stability. We validated an antibody that recognizes both human and mouse VEGFR1 (FLT-1) using wild-type and *Flt-1*^*−/−*^ mouse ES cells, and detected a band at 180 kD in wild-type but not mutant ES cells (Supplementary Fig. 1a), indicating specific detection of mVEGFR1. We next assessed baseline turnover of mVEGFR1 and VEGFR2 using primary human umbilical vein endothelial cells (HUVEC) cultured with cycloheximide (CHX) to inhibit new protein synthesis. A dose-response analysis revealed no loss of mVEGFR1 while VEGFR2 was undetectable at all doses (Fig. 1a). A time course using 0.5 mg ml^−1^ of CHX showed that VEGFR2 was undetectable after 2 h of CHX treatment (Fig. 1b), consistent with a reported half-life of 70 min (ref. 36). Surprisingly, mVEGFR1 was not reduced with time, even after 28 h, in either HUVEC or another human endothelial cell, HBMVEC (human brain microvascular endothelial cells) (Fig. 1b,c). We verified VEGFR1 baseline stability by quantitative immunofluorescence, which does not distinguish VEGFR1 isoforms, but nevertheless showed no significant reduction in VEGFR1 signal over 24 h (Supplementary Fig. 1b). These findings indicate that mVEGFR1 turns over very slowly (half-life >28 h) in relation to VEGFR2 in endothelial cells.

We utilized a 3D sprouting angiogenesis assay to test VEGFR1/2 turnover in a more physiologically relevant setting^37^. Endothelial cells sprout from beads embedded in fibrin to form polarized sprouts with patent lumens over a 6-day period. HUVEC sprouts treated with CHX for 18 h had no detectable VEGFR2 staining, but about half of the VEGFR1 signal remained (Fig. 1d,e), suggesting that VEGFR1 is significantly more stable than VEGFR2 in 3D sprouts. Taken together, the two receptors have very different turnover kinetics, consistent with their different roles in modulating VEGF-A signalling and angiogenesis.

We next investigated the trafficking of mVEGFR1, and asked whether the receptor reaches the cell surface and constitutively internalizes from the surface. HUVEC were incubated with an N-terminal-specific antibody to VEGFR1 and either the dynamin inhibitor dynasore to block internalization, or the VEGFR1-specific-ligand PLGF to promote internalization. Immunofluorescence revealed localized puncta of VEGFR1 that were dynamin-dependent (Fig. 1f). These results suggest that VEGFR1 resides at the cell surface and constitutively internalizes, perhaps via clathrin-coated pits, although other routes cannot be ruled out. The VEGFR1 puncta were also observed in PLGF-stimulated HUVEC, consistent with ligand-stimulated internalization. We confirmed that VEGFR1 was surface labelled and internalized by stripping surface-bound antibodies after allowing for internalization^38^. VEGFR1 puncta formed at 37 °C were resistant to the stripping protocol, while cells held at 4 °C to block internalization were sensitive to stripping (Supplementary Fig. 1c,d), indicating surface localization and constitutive internalization.

Early endosomes sort internalized cargo for recycling or degradation, and proteins typically recycle from endosomes to the cell surface in a Rab4a-dependent (fast) or Rab11a-dependent (slow) manner^39^. To distinguish these possibilities, we disrupted recycling via siRNA-mediated knockdown of Rabs in HUVEC. mVEGFR1 turnover was unaffected in control and Rab4 knockdown endothelial cells with new protein synthesis inhibited, while about 70% of mVEGFR1 was lost with Rab11a knockdown (Supplementary Fig. 1e, Fig. 1g). Immunofluorescence showed that 90% of the VEGFR1 signal was lost with Rab11a knockdown (Fig. 1h,i). Taken together, these results indicate that mVEGFR1 constitutively internalizes and suggest Rab11a-dependent recycling to the plasma membrane, which is consistent with its long half-life and non-signalling function during vascular sprouting.

### Palmitoylation dynamically regulates VEGFR1 stability

**Figure 2:**
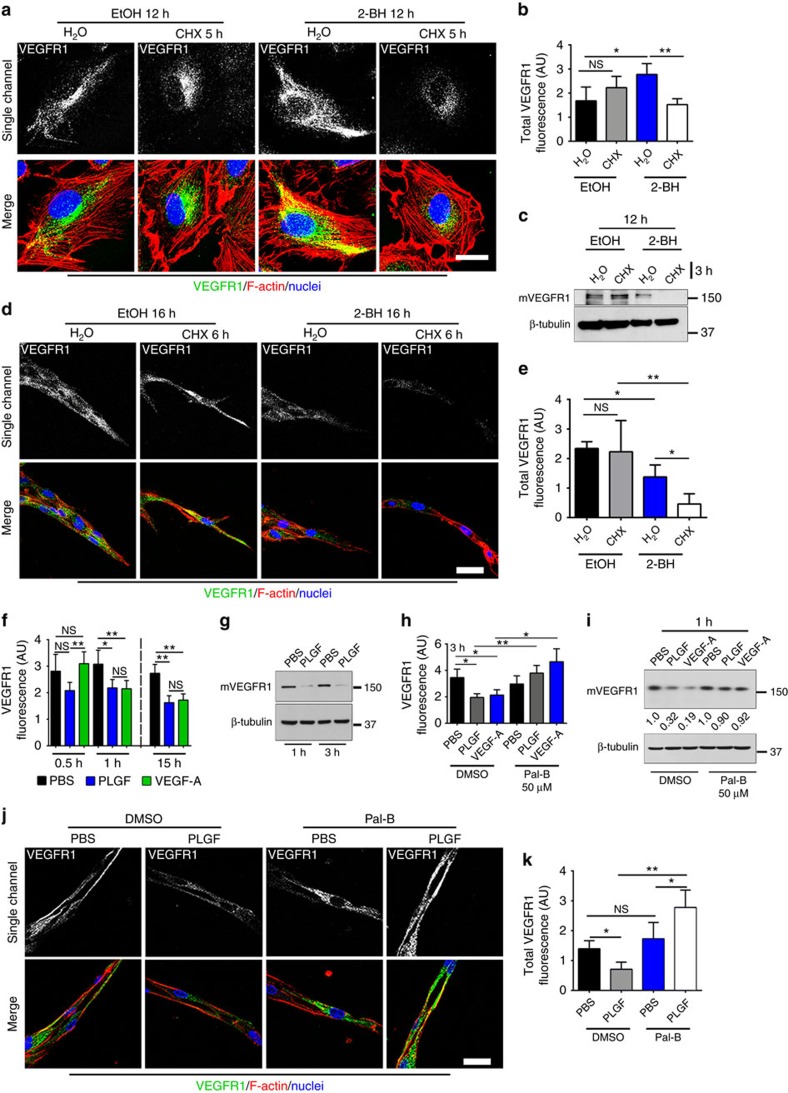
Palmitoylation regulates mVEGFR1 stability. (**a**,**b**) (**a**) VEGFR1 immunofluorescence of HUVEC with indicated treatments. (**b**) Quantification of fluorescence via integrated density. (No. of cells: EtOH/H_2_O, *n*=20; EtOH/CHX, *n*=12; 2-BH/H_2_O *n*=24; 2-BH/CHX *n*=18); CHX, cycloheximide; two replicates, scale bar: 25 μm. (**c**) Immunoblot of HUVEC with indicated treatments; four replicates. (**d**,**e**) (**d**) VEGFR1 immunofluorescence of d5 HUVEC angiogenic sprouts with indicated treatments; 2-BH, 2-bromohexadecadnoic acid, scale bar: 25 μm. (**e**) Quantification of fluorescence via integrated density. (No. of sprouts: EtOH/H_2_O, *n*=20; EtOH/CHX, *n*=12; 2-BH/H_2_O, *n*=24; 2-BH/CHX, *n*=18); two replicates. (**f**) Quantification of VEGFR1 fluorescence in HUVEC with indicated treatments via integrated density. (*n*=50 cells per condition); four replicates. (**g**) Immunoblot of HUVEC stimulated with 50 ng ml^−1^ PLGF or PBS for indicated times; four replicates. (**h**) Quantification of VEGFR1 fluorescence of HUVEC with indicated treatments and 50 ng ml^−1^ PLGF or VEGF-A via integrated density. Pal-B, palmostatin-B. (No. of cells: DMSO/PBS, *n*=51; DMSO/PLGF, *n*=37; DMSO/VEGF-A, *n*=45; Pal-B/PBS, *n*=50; Pal-B/PLGF *n*=48; Pal-B/VEGF-A, *n*=51); three replicates. (**i**) Immunoblot of HUVEC with indicated treatments and 50 ng ml^−1^ PLGF or VEGF-A for 1 h. Values are mVEGFR1 normalized to respective PBS controls; three replicates. (**j**,**k**) (**j**) VEGFR1 immunofluorescence of d5 HUVEC angiogenic sprouts with indicated treatments and 75 ng ml^−1^ PLGF or PBS for 4 h, scale bar: 25 μm. (**k**) Quantification of fluorescence via integrated density. (No. of sprouts: DMSO/PBS *n*=13; DMSO/PLGF, *n*=17; Pal-B/PBS *n*=12; Pal-B/PLGF *n*=16); two replicates. Statistics: Shown are means+95% CI. One-way ANOVA with pairwise comparison and post-hoc Tukey's range test. *P≤0.05; **P≤0.01; NS, not significant.

Palmitoylation dynamically regulates protein trafficking and turnover^29^. Thus, we hypothesized that palmitoylation regulates VEGFR1 stability. Since mVEGFR1 has several potential palmitoylation sites based on PalmPred palmitoylation^40^ and CSS Palm 2.0 (ref. 41) prediction algorithms, we determined effects of manipulating palmitoylation on baseline turnover of mVEGFR1 by inhibiting palmitoylating enzymes (protein acetyl-transferases) with 2-bromohexadecadnoic acid (2-BH). Palmitoylation blockade in HUVEC caused rapid turnover of VEGFR1 upon blockade of new protein synthesis (Fig. 2a,b), and immunoblot confirmed the membrane-bound VEGFR1 isoform was affected (Fig. 2c), suggesting that palmitoylation stabilizes mVEGFR1. Similarly, 2-BH-induced palmitoylation blockade significantly decreased baseline stability of VEGFR1 in angiogenic sprouts (Fig. 2d,e), and levels were rescued by inhibiting the lysosome with chloroquine in angiogenic sprouts (Supplementary Fig. 2a,b), implying that palmitoylation stabilizes mVEGFR1 by preventing its lysosomal degradation.

Ligand-bound VEGFR1 is internalized and degraded^26^. Incubation of HUVEC with VEGF-A or PLGF led to significant loss of mVEGFR1, although PLGF incubation reduced VEGFR1 levels more quickly than did VEGF-A (Fig. 2f,g). PLGF-induced degradation of mVEGFR1 was blocked by inclusion of chloroquine, but not the proteasome inhibitor MG132 (Supplementary Fig. 2c,d), demonstrating that PLGF binding promotes lysosomal degradation of mVEGFR1. PLGF- or VEGF-A-induced loss of mVEGFR1 was blocked by Palmostatin-B (Pal-B), an inhibitor of depalmitoylating enzymes (acyl-protein thioesterases, APTs) (Fig. 2h,i). We also found that blockade of depalmitoylation prevented ligand-induced loss of VEGFR1 in angiogenic spouts (Fig. 2j,k), consistent with the 2D data. Collectively, these results show that palmitoylation regulates the stability and ligand-induced turnover of mVEGFR1 in endothelial cells and angiogenic sprouts.

### mVEGFR1 palmitoylation regulates trafficking

**Figure 3:**
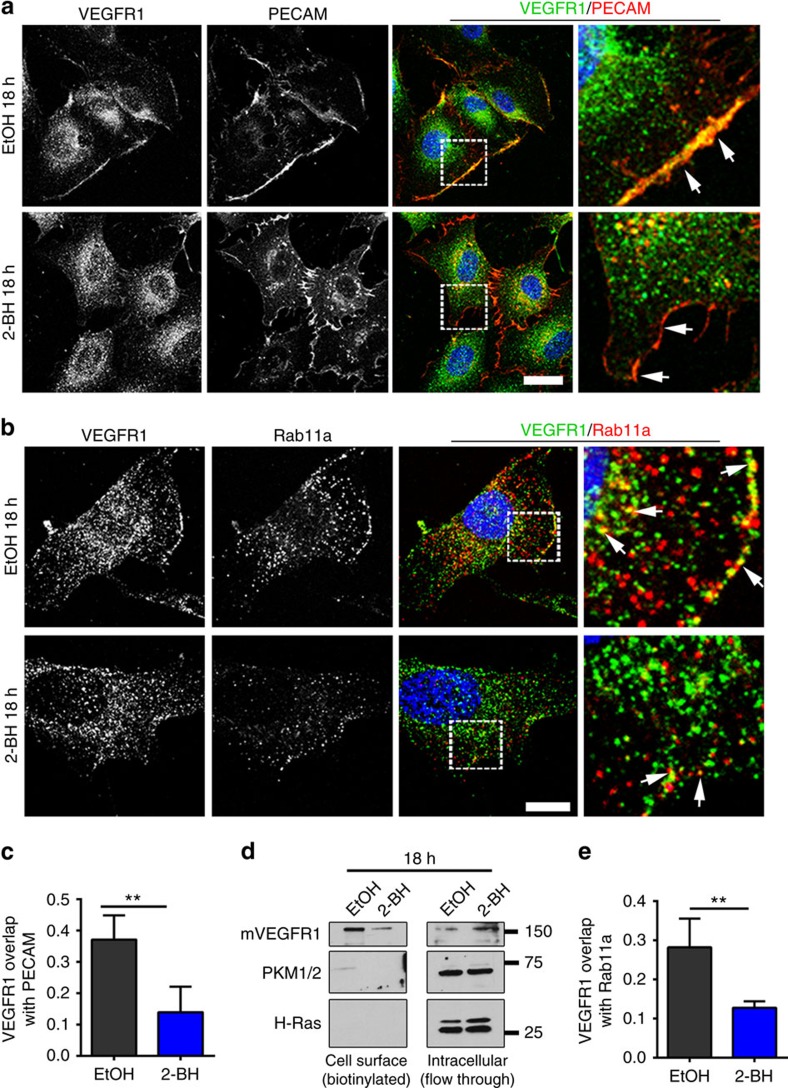
Palmitoylation regulates VEGFR1 trafficking. (**a**,**b**) (**a**) Immunofluorescence of VEGFR1 and PECAM or (**b**) VEGFR1 and Rab11a in HUVEC with indicated treatments; 2-BH, 2-bromohexadecadnoic acid, scale bars: 20 and 10 μm for **a**,**b**, respectively. (**c**,**e**) Mander's Correlation Coefficient quantification of overlap of VEGFR1 and PECAM (**c**) or Rab11a (**e**) in HUVEC treated as indicated. (No. of cells: VEGFR1/PECAM: EtOH, *n*=14; 2-BH, *n*=12; VEGFR1/Rab11: EtOH, *n*=23; 2-BH, *n*=27); three replicates. (**d**) Immunoblot of cell surface or intracellular VEGFR1 from HUVEC with indicated treatments. PKM1/2, cytoplasmic marker; H-Ras, specificity control. Statistics: Shown are means+95% CI. Student's *t*-test with post-hoc Tukey's range test. **P≤0.01.

To determine the effects of VEGFR1 palmitoylation on its trafficking, we examined localization under conditions of impaired activity. We first examined the co-localization of an endothelial cell surface marker, PECAM-1 (PECAM) with VEGFR1. There was measurable overlap at baseline, consistent with the idea that some mVEGFR1 is cell-surface localized, and this overlap was significantly reduced upon blockade of palmitoylation with 2-BH (Fig. 3a,c). Immunoblot analysis after cell surface biotinylation confirmed that blockade of palmitoylation reduced cell surface-localized mVEGFR1 and increased its intracellular location, consistent with palmitoylation being involved in residence of mVEGFR1 at the cell surface (Fig. 3d). We next examined the co-localization of VEGFR1 and Rab11a and found that overlap that was significantly reduced with blockade of palmitoylation, in line with a role for Rab11a in regulating VEGFR1 recycling (Fig. 3b,e). Finally, we hypothesized that blockade of palmitoylation would increase the amount of lysosome-mediated degradation of VEGFR1, and consistent with this idea found significantly increased co-localization of VEGFR1 and the lysosome marker LAMP-1 upon palmitoylation blockade (Supplementary Fig. 3a,b). These data show that post-translational palmitoylation of mVEGFR1 likely mediates trafficking to the cell surface and slow recycling.

### mVEGFR1 palmitoylation is enzymatically regulated

**Figure 4:**
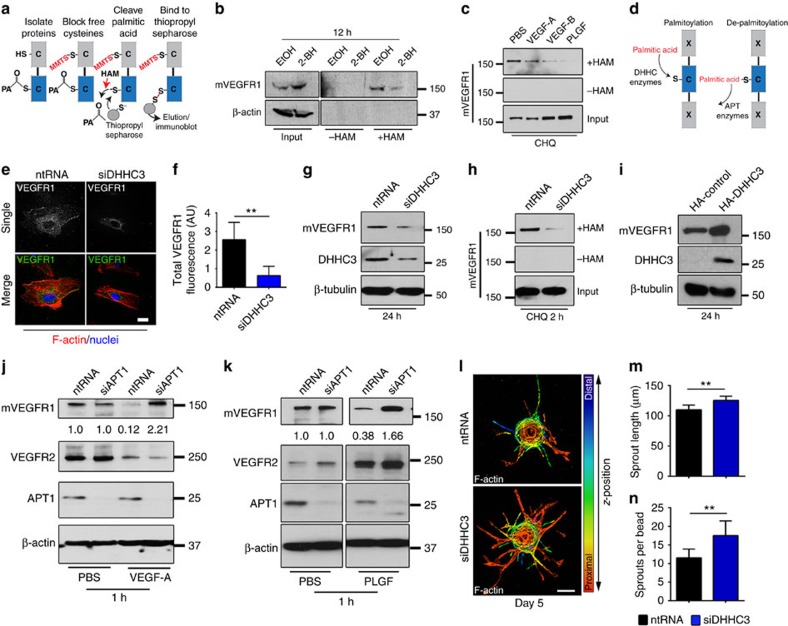
VEGFR1 is palmitoylated in endothelial cells to regulate turnover. (**a**) Workflow for acyl-resin assisted capture (Acyl-RAC). PA, palmitic acid; MMTS, methyl-methanethiosulfonate. (**b**) Acyl-RAC and immunoblot of HUVEC with indicated treatments. 2-BH, 2-bromohexadecadnoic acid; three replicates. (**c**) Acyl-RAC and immunoblot of VEGFR1 from HUVEC with indicated treatments for 45 min. CHQ, chloroquine; three replicates. (**d**) Diagram illustrating palmitic acid addition and removal. DHHC, protein acetyl-transferase; APT, acyl-protein thioesterase. (**e**,**f**) (**e**) VEGFR1 immunofluorescence of HUVEC with indicated treatments for 24 h, scale bar: 25 μm. (**f**) Quantification of fluorescence via integrated density. Shown are means+95% CI. (No. of cells: ntRNA, *n*=21; siDHHC3, *n*=22); four replicates. (**g**) Immunoblot of mVEGFR1 from HUVEC with indicated treatments; three replicates. (**h**) Acyl-RAC and immunoblot of HUVEC with indicated treatments for 24 h; three replicates. (**i**) Immunoblot of VEGFR1 and HA-tagged DHHC3 in HUVEC with indicated treatments for 24 h; three replicates. (**j**,**k**) Immunoblot of HUVEC with indicated treatments at 48 h post knockdown and treated with 50 ng ml^−1^ VEGF-A (**j**) or PLGF (**k**); three replicates. (**l**–**n**) (**l**) HUVEC angiogenic sprouts with indicated treatments, scale bar: 100 μm. (**m**,**n**) Quantification of angiogenic parameters. (sprouts per bead no. of beads: ntRNA, *n*=25; siDHHC3, *n*=27; sprout length, no. of sprouts: ntRNA, *n*=288; siDHHC3, *n*=365); two replicates. Statistics: Students *t*-test and post-hoc Tukey's range test. **P≤0.01.

To directly assess palmitoylation of mVEGFR1 in endothelial cells, we utilized an acyl-resin-assisted capture (Acyl-RAC) assay^42^. Blockade of free cysteines, followed by hydroxylamine (HAM)-mediated cleavage of palmitic acid and protein capture with thiopropyl-sepharose revealed mVEGFR1 capture that was reduced by inclusion of the palmitoylation inhibitor 2-BH relative to controls, suggesting that mVEGFR1 is palmitoylated in endothelial cells (Fig. 4a,b). Palmitoylation of mVEGFR1 in endothelial cells was supported using another assay, Acyl-biotin exchange^43^ (Supplementary Fig. 4a,b). We next hypothesized that depalmitoylation of mVEGFR1 is required for ligand-induced instability, and used the Acyl-RAC assay to reveal reduced levels of captured mVEGFR1 upon incubation with the VEGFR1 ligands VEGF-A, VEGF-B or PLGF, relative to controls (Fig. 4c), indicating that ligand binding reduces steady-state levels of palmitoylated mVEGFR1.

DHHC enzymes palmitoylate and APT enzymes depalmitoylate proteins^44^,^45^ (Fig. 4d). Because DHHC3 is expressed and functionally active in endothelial cells^46^, we hypothesized that it regulates baseline mVEGFR1 levels. We confirmed reduced DHHC3 protein with siRNA knockdown (Fig. 4g, Supplementary Fig. 4c,d), and found that DHHC3 knockdown in HUVEC significantly lowered baseline mVEGFR1 levels (Fig. 4e–g, Supplementary Fig. 4d) and reduced mVEGFR1 captured by the Acyl-RAC assay compared to controls, suggesting reduced palmitoylation of the receptor (Fig. 4h). In contrast, reduction of another palmitoylating enzyme expressed in endothelial cells, DHHC7, did not affect mVEGFR1 levels (Supplementary Fig. 4e), while over-expression of DHHC3 increased levels of mVEGFR1 in endothelial cells (Fig. 4i). Thus, DHHC3 regulates mVEGFR1 stability in endothelial cells, perhaps via direct palmitoylation of mVEGFR1.

We next asked whether loss of the depalmitoylating enzyme APT1 prevented ligand-induced degradation of mVEGFR1, and found that APT1 knockdown along with incubation with either VEGF-A or PLGF blocked loss of mVEGFR1 and caused upregulation of mVEGFR1 levels, perhaps as a result of impaired turnover (Fig. 4j,k). In contrast, APT1 knockdown did not significantly alter VEGFR2 levels, although downregulation of VEGFR2 with VEGF-A and upregulation with PLGF treatment was observed, as has been reported in other systems^47^. We next assessed the effects of DHHC3 knockdown on sprouting angiogenesis, and showed increased sprouting with reduced DHHC3 levels (Fig. 4l–n). Thus, the proximate regulators of mVEGFR1 palmitoylation and stability in endothelial cells are likely DHHC3 and APT1.

### Rab27a regulates DHHC3 localization and VEGFR1 stability

**Figure 5:**
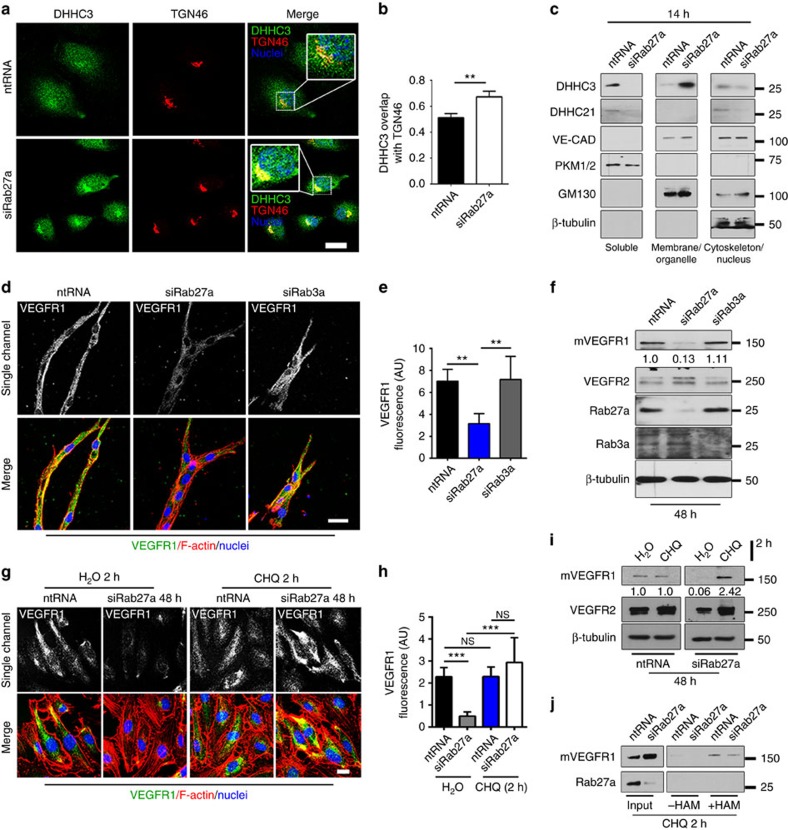
Rab27a regulates DHHC3 localization and palmitoylation of VEGFR1. (**a**,**b**) DHHC3 and TGN46 (Golgi) immunofluorescence (**a**) and Mander's Correlation Coefficient quantification (**b**) of overlap in HUVEC with indicated treatments; three replicates, scale bar: 20 μm. (**c**) Subcellular fractionation and immunoblot of HUVEC with indicated treatments. Pyruvate kinase1/2 (PKM1/2), soluble marker; vascular-endothelial cadherin (VE-CAD), membrane and cytoskeletal marker; cis-Golgi Marker 130 (GM130), membrane marker; two replicates. (**d**,**e**) (**d**) VEGFR1 immunofluorescence of d4 HUVEC angiogenic sprouts with indicated siRNAs, scale bar: 25 μm. (**e**) Quantification of fluorescence via integrated density. (No. of sprouts: ntRNA, *n*=7; siRab27a, *n*=9; siRab3a, *n*=5); three replicates. (**f**) Immunoblot of HUVEC with indicated treatments. Values are relative mVEGFR1 levels. Doublet in VEGFR2 lane is likely due to glycosylation; four replicates. (**g**,**h**) (**g**) VEGFR1 immunofluorescence of HUVEC with indicated treatments, scale bar: 15 μm. (**h**) Quantification of fluorescence via integrated density. (No. of cells: ntRNA, H_2_O, *n*=28; CHQ, *n*=22; siRab27a, H_2_O, *n*=13; CHQ, *n*=15); two replicates. (**i**) Immunoblot of HUVEC with indicated treatments. Values are relative mVEGFR1 levels; 3 replicates. (**j**) Acyl-RAC and immunoblot of HUVEC with indicated siRNAs; three replicates. Statistics: Shown are means +95% CI. One-way ANOVA with pairwise comparison and post-hoc Tukey's range test (**e**,**h**); student's *t*-test (**b**). **P≤0.01; ***P≤0.001; NS, not significant.

To better understand regulation of VEGFR1 turnover, we considered potential upstream regulators of DHHC3, and we hypothesized that spatial regulation of enzyme availability to mVEGFR1 is coordinated by Rab GTPases. Although numerous Rab GTPases affect receptor trafficking^48^,^49^, little is known about direct effects on stability via effects on post-translational modifiers. Because Rab27a regulates protein stability and trafficking^50^,^51^ and is active in endothelial cells, we hypothesized that Rab27a regulates proper trafficking of palmitoylation enzymes. When Rab27a levels were reduced by knockdown in endothelial cells, DHHC3 overlap with the Golgi marker TGN46 increased and overlap with Rab27a decreased in the absence of reduced DHHC3 levels, consistent with mis-trafficking (Fig. 5a,b, Supplementary Fig. 5a–c). In contrast, reduction of Rab11a did not alter DHHC3 overlap with TGN46, showing specificity for effects of Rab27a knockdown and suggesting that palmitoylation is upstream of Rab11a trafficking of mVEGFR1 (Supplementary Fig. 5d). Based on increased localization of DHHC3 to the Golgi with reduced Rab27a, we hypothesized that Rab27a regulates the spatial distribution of DHHC3 in endothelial cells. Sub-cellular fractionation showed that DHHC3 localized to the soluble fraction and, to a lesser extent, the cytoskeletal fraction in controls. Strikingly, DHHC3 was undetectable in the soluble and cytoskeletal fractions in endothelial cells with reduced Rab27a levels, but accumulated to high levels in the membrane fraction that is also enriched for the Golgi marker GM130 (Fig. 5c). Consistent with our hypothesis, Rab27a was also localized to the soluble and cytoskeletal fractions (Supplementary Fig. 5e). DHHC21, another acetyl-transferase expressed in endothelial cells, did not follow those trends in response to reduced Rab27a levels. These findings are consistent with a mechanism whereby the spatial distribution of DHHC3 is regulated by Rab27a.

These findings suggested that reduced levels of Rab27a might destabilize mVEGFR1 as a result of impaired palmitoylation downstream of DHHC3 mis-trafficking. Consistent with this hypothesis, there was little overlap between Rab27a and VEGFR1 in endothelial cells by immunofluorescence analysis (Supplementary Fig. 5f), but substantial overlap of VEGFR1 and DHHC3 that was significantly decreased with reduced Rab27a levels (Supplementary Fig. 6a–c). We next assessed VEGFR1 levels in angiogenic sprouts or cultured endothelial cells after knockdown of Rab27a or Rab3a, another protein trafficking regulator that has some overlapping functions with Rab27a (ref. 52). VEGFR1 was significantly reduced in Rab27a knockdown sprouts and by immunoblot, while knockdown of Rab3a did not affect VEGFR1 levels in sprouts or by immunoblot (Fig. 5d–f, Supplementary Fig. 6d). In contrast, VEGFR2 levels remained unchanged in Rab27a knockdown sprouts and were marginally elevated in immunoblots of endothelial cells (Fig. 5f, Supplementary Fig. 6e,f). These results imply that Rab27a regulates mVEGFR1 protein levels in endothelial cells. To determine the mechanism of mVEGFR1 degradation in response to Rab27a knockdown, HUVEC with reduced Rab27a or controls were exposed to lysosome or proteasome inhibitors. Chloroquine (lysosome) completely rescued mVEGFR1 in Rab27a knockdown HUVEC (Fig. 5g–i), while MG132 (proteasome) did not rescue but significantly downregulated baseline mVEGFR1 levels (Supplementary Fig. 6g–i). These findings indicate that Rab27a regulates mVEGFR1 protein levels by preventing lysosome-mediated degradation of the receptor in endothelial cells.

We hypothesized that Rab27a-mediated mVEGFR1 stability was linked to palmitoylation. To test this, we performed the Acyl-RAC assay after incubating HUVEC with chloroquine to restore mVEGFR1 levels in the presence of reduced Rab27a levels. Consistent with mis-localized DHHC3, mVEGFR1 capture was significantly reduced in HUVEC with reduced Rab27a (Fig. 5j), suggesting reduced palmitoylation. Collectively, these data show that Rab27a regulates mVEGFR1 levels and suggest that it functions through modulation of receptor palmitoylation, likely via spatial regulation of DHHC3.

### Rab27a is a negative regulator of angiogenesis

**Figure 6:**
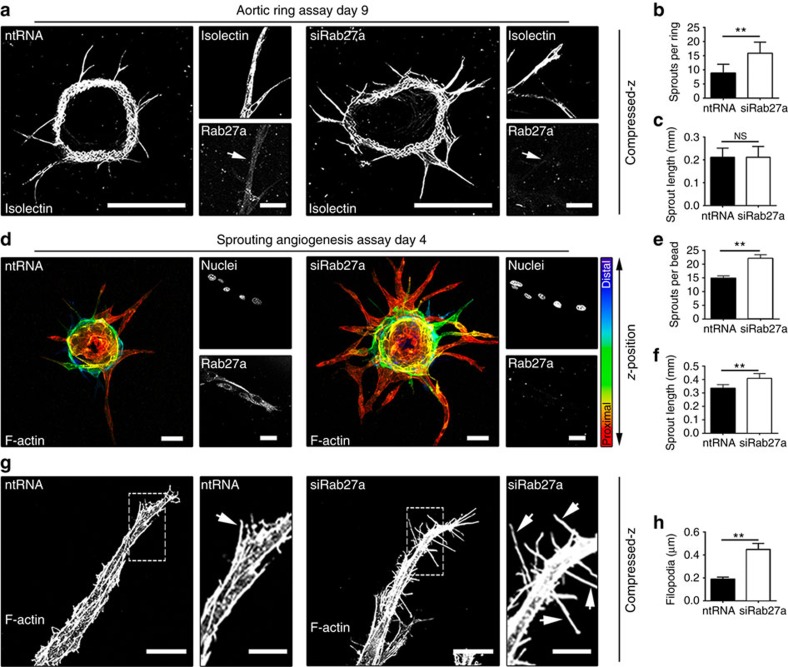
Rab27a regulates vascular sprouting. (**a**–**c**) (**a**) Aortic rings with indicated siRNAs. Small boxes, indicated stainings, scale bars: 500 and 25 μm for large and small boxes, respectively. (**b**,**c**) Quantification of angiogenic parameters after indicated treatments. (No. of rings, ntRNA=14; siRab27a=12); three replicates. (**d**–**f**) (**d**) HUVEC angiogenic sprouts with indicated treatments. Small boxes, indicated stainings, scale bars 50 and 25 μm for large and small boxes, respectively. (**e**,**f**) Quantification of angiogenic parameters. (sprouts per bead: ntRNA=12; siRab27a=14; sprout length: ntRNA=30; siRab27a=30); five replicates. (**g**,**h**) (**g**) Morphological analysis of HUVEC derived-sprouts with indicated treatments, scale bars: 25 and 10 μm, for large and small boxes, respectively. (**h**) Quantification of filopodia per μm of vessels. Arrows, filopodia. (ntRNA=10; siRab27a=14); five replicates. Statistics: Shown are means +95% CI. (**b**,**c**,**e**,**f**,**h**) Student's *t*-test. **P≤0.01; NS, not significant.

VEGFR1 negatively regulates blood vessel growth *in vitro* and *in vivo*, but no regulatory function for Rab27a during angiogenesis has been described. We hypothesized that Rab27a is anti-angiogenic via its role in stabilizing mVEGFR1. Rab27a knockdown in *ex vivo* mouse aortic rings^53^ significantly increased angiogenic sprouting, although overall sprout length was not affected (Fig. 6a–c). Rab27a knockdown in the sprouting angiogenesis assay using HUVEC increased angiogenic sprouting and sprout length, consistent with its hypothesized anti-angiogenic function (Fig. 6d–f). Time course analysis showed that Rab27a knockdown HUVEC had increased sprouts at all times, while Rab3a knockdown did not affect angiogenic sprouting (Supplementary Fig. 7a–d). Filopodia are thin, cellular actin-rich extensions that sense the environment, influence endothelial cell migration and are considered a hallmark of ‘active' endothelium. Analysis of Rab27a knockdown HUVEC sprouts also revealed a significant increase in filopodia (Fig. 6g,h). Thus, knockdown of Rab27a leads to increased HUVEC sprouting, suggesting a novel anti-angiogenic function for Rab27a, likely through regulation of mVEGFR1 stability.

### Angiogenic effects of Rab27a are epistatic to VEGFR1

**Figure 7:**
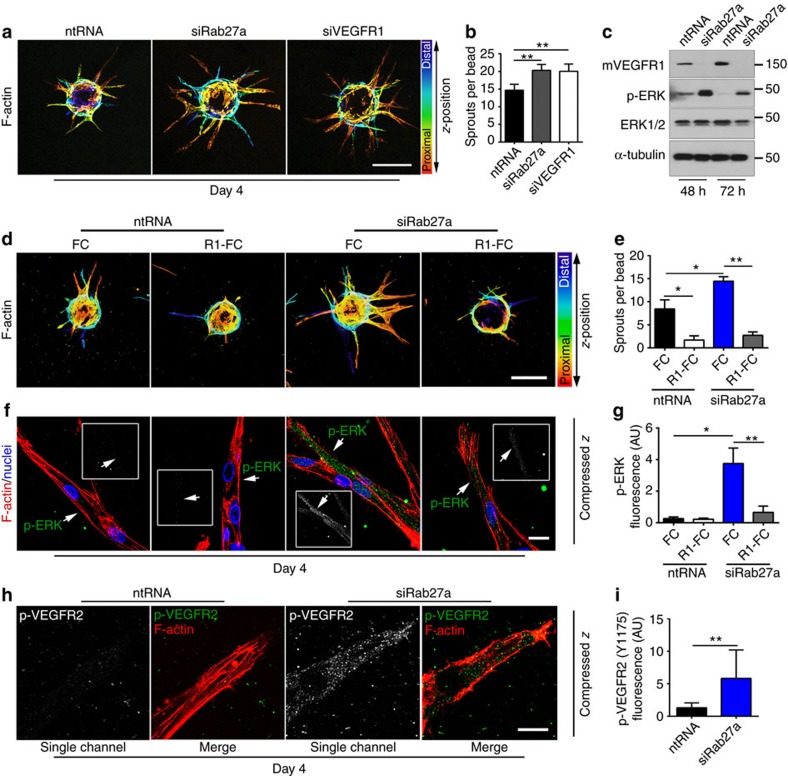
VEGFR1 is epistatic to Rab27a. (**a**,**b**) (**a**) HUVEC angiogenic sprouts and (**b**) quantification of sprouting after indicated treatments, scale bar: 200 μm. (sprouts per bead, No. of beads: ntRNA=18; siRab27a=15; siVEGFR1=16); three replicates. (**c**) Immunoblot of HUVEC with indicated treatments; three replicates. (**d**–**g**) (**d**) HUVEC angiogenic sprouts with indicated treatments, scale bar: 200 μm. FC, control; R1-FC, recombinant human VEGFR1. (**e**) Quantification of sprouts per bead. (**f**) pERK immunofluorescence of HUVEC angiogenic sprouts with indicated treatments, scale bar: 20 μm. (**g**) pERK quantification via integrated density. (pERK fluorescence, No. of sprouts: ntRNA/FC=8; ntRNA/R1-FC=9; siRab27a/FC=10; siRab27a/R1-FC=7; sprouts per bead, No. of beads: ntRNA/FC=14; ntRNA/R1-FC=14; siRab27a/FC=12; siRab27a/R1-FC=15); three replicates. (**h**,**i**) (**h**) pVEGFR2 (Y1175) Immunofluorescence of HUVEC angiogenic sprouts with indicated treatments, scale bar: 10 μm. (**i**) pVEGFR2 quantification via integrated density. (No. of sprouts: ntRNA=9; siRab27a=9); three replicates. Statistics: Shown are means +95% CI. One-way ANOVA and pairwise comparison with post-hoc Tukey's range test. *P≤0.05; **P≤0.01.

We wondered whether Rab27a function is epistatic to mVEGFR1 in sprouting angiogenesis. We found that reduced VEGFR1 levels (Supplementary Fig. 7e) phenocopied the increased sprouting seen in Rab27a knockdown sprouts, with no distinguishable differences in sprout morphology or branching (Fig. 7a,b). Because VEGFR1 negatively modulates signalling through VEGFR2 (refs 21, 54) and ERK activation is a downstream effector of VEGFR2 signalling, we assessed pERK levels after Rab27a knockdown in endothelial cells and found a significant increase in both pERK and phosphorylated histone-H3, a mitosis marker downstream of ERK activation (Fig. 7c, Supplementary Fig. 7f,g).

We next asked whether exogenous addition of VEGFR1 altered the effects of reduced Rab27a levels on sprouting angiogenesis, using a truncated form of VEGFR1 linked to an FC domain (R1-FC), and found that the excess sprouting seen with reduced Rab27a was significantly reduced by exogenous R1-FC (Fig. 7d,e). These findings indicate that reduced Rab27a cannot overcome the negative effects of exogenous VEGFR1 on sprouting and suggest that they act through similar mechanisms. Further support for this hypothesis is that R1-FC significantly blunted the elevated pERK levels found in sprouts with reduced Rab27a (Fig. 7f,g). We also observed increased pVEGFR2 in angiogenic sprouts with reduced Rab27a (Fig. 7h,i), suggesting that increased ERK activation in Rab27a knockdown sprouts is downstream of elevated VEGFR2 signalling. Collectively, these results imply that loss of mVEGFR1 downstream of Rab27a knockdown is required for the effects of reduced Rab27a on angiogenesis, likely through increased VEGFR2 signalling and activation of downstream effector pathways.

### Rab27a regulates FLT-1 levels and angiogenesis *in vivo*

**Figure 8:**
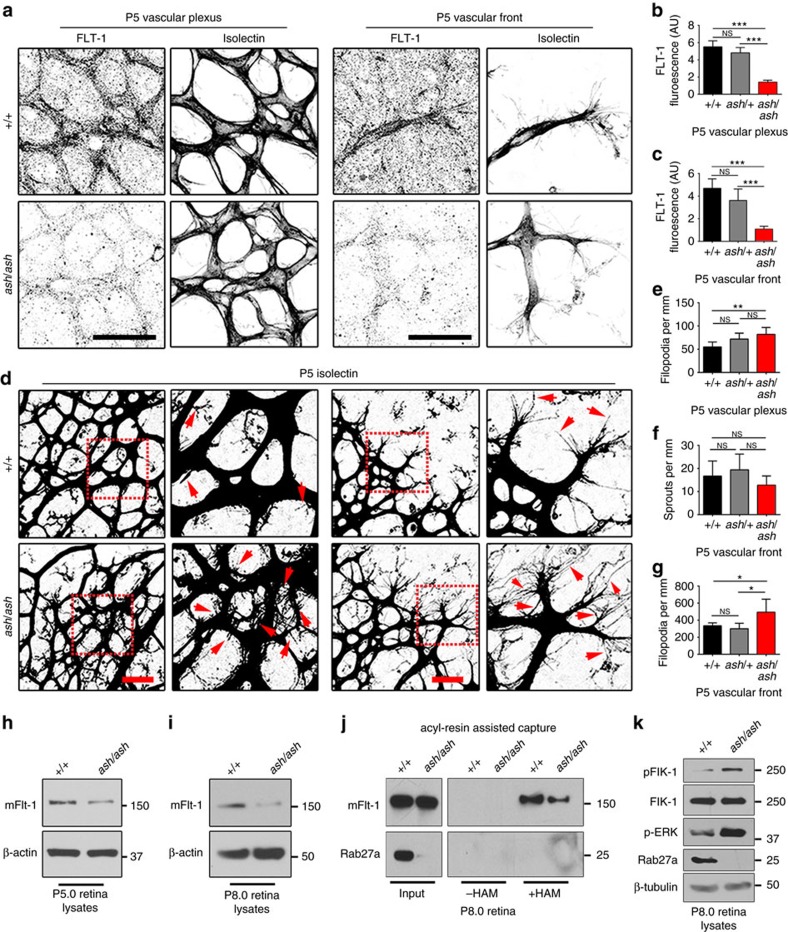
*Ashen* retinal vessels have reduced FLT-1 and excess filopodia. (**a**–**c**) (**a**) FLT-1 immunofluorescence of retinas from P5 mice with indicated genotypes, scale bars: 50 μm. (**b**,**c**) Quantification of FLT-1 in indicated areas via integrated density. Scale bar, 25 μm. (No. of retinas: +/+, *n*=4; *ash*/+, *n*=6; *ash/ash, n*=4). (**d**–**g**) (**d**) Immunofluorescence of retinas. Quantification of (**e**) filopodia in the plexus (**f**) sprouting, and (**g**) filopodia at the vascular front from P5 mice with indicated genotypes. Red box, magnified at right; arrows, filopodia. Scale bars: 25 μm. (No. of retinas from two independent litters: +/+, *n*=6, *ash*/+ *n*=5; *ash/ash, n*=7). (**h**,**i**) Immunoblot for mFLT-1 in retinal lysates from indicated genotypes at P5 (**h**) and P8 (**i**); four replicates per stage. (**j**) Acyl-RAC and immunoblot for mFLT-1 and Rab27a from retinal lysates at indicated stages; three replicates. (**k**) Immunoblot of retinal lysates as indicated; three replicates. Statistics: Shown are means +95% CI. One-way ANOVA and pairwise comparison with post-hoc Tukey's range test. *P≤0.05; **P≤0.01; ***P≤0.001; NS, not significant.

*Ashen* mice have a spontaneous point mutation in the *rab27a* locus leading to loss-of-function^55^. Mice homozygous for *ashen* (*ash/ash*) are viable and have several defects linked to mis-trafficking, including defective melanosome trafficking that leads to the ‘ash-like' coat colour. We hypothesized that blood vessels of *ash/ash* mice had angiogenic defects resulting from destabilized FLT-1 (mouse VEGFR1). *Ashen* mice are on the C3H/HeSn genetic background that also carries the *rd* mutation, leading to retinal degeneration starting at 2–3 weeks of age^56^,^57^. Thus we analysed post-natal retinal vessels that expand via sprouting angiogenesis during the first week of life, prior to photoreceptor cell degradation, and we compared all effects to littermate controls that also carried the *rd* mutation. We found detectable levels of Rab27a in vessels at the vascular front and in the vascular plexus behind the front in wild-type controls (+/+) at post-natal day 6.5 (P6.5) that were dramatically reduced in *ash/ash* homozygous littermates, along with undetectable levels of Rab27a in *ash/ash* retinal lysates (P6.0) (Supplementary Fig. 8a,b). Consistent with the hypothesis that loss of Rab27a destabilizes VEGFR1 *in vivo*, post-natal (P5) retinal vessels of *ash/ash* homozygous mice had significantly reduced levels of FLT-1 staining at the vascular front and in the plexus behind the front compared to littermate controls (Fig. 8a–c). Examination of vascular morphology showed significantly increased filopodia in the plexus vessels and at the vascular front of *ash/ash* retinas at P5, although overall sprout numbers were not affected (Fig. 8d–g, inlay, red arrows). We also analysed later stages (Supplementary Fig. 8c–h) and found that the increased filopodia numbers were resolved at the vascular front by P6.5, but persisted in the plexus vessels through P8.5. Consistent with these findings, filopodia length was also increased in P8.5 retinas in the vascular plexus but not at the vascular front (Supplementary Fig. 9a–c), suggesting that loss of Rab27a in retinal vessels results in an initial hyperactive phenotype that perdures in the normally more quiescent plexus behind the front with time. However, vessel density was not increased in *ash/ash* mutant retinas at P5.0–8.5 (Supplementary Fig. 9d–f), suggesting that hyperactivity of *ash/ash* vessels leads to temporary morphogenetic changes that are compensated *in vivo*.

We next asked whether the biochemical changes to VEGFR1 and VEGF-A signalling components documented *in vitro* were seen *in vivo*. We analysed retinal lysates and found reduced levels of mFLT-1 at both P5.0 and P8.0 in *ash/ash* retinas compared to wild-type littermate controls (Fig. 8h,i), consistent with the hypothesis that mFLT-1 is destabilized with loss of Rab27a *in vivo*. We performed the Acyl-RAC assay and found reduced capture of mFLT-1 in p8.0 retinal lysates of *ash/ash* retinas compared to controls (Fig. 8j, Supplementary Fig. 10), indicating that mFLT-1 palmitoylation is compromised *in vivo* with loss of Rab27a. The retinal lysates were also analysed for VEGF-A signalling changes, and *ash/ash* retinas had elevated pFlk-1 and pERK relative to controls (Fig. 8k), suggesting that the decoy function of mFLT-1 in VEGF-A signalling regulation is compromised *in vivo* with loss of Rab27a. Collectively, these findings show that Rab27a regulates FLT-1 levels during angiogenesis *in vivo*, and reveal a vascular phenotype in *ash/ash* mice that is consistent with destabilization of mFLT-1, presumably via impaired palmitoylation and increased turnover of the receptor, and increased VEGF-A signalling downstream of these effects.

## Discussion

**Figure 9:**
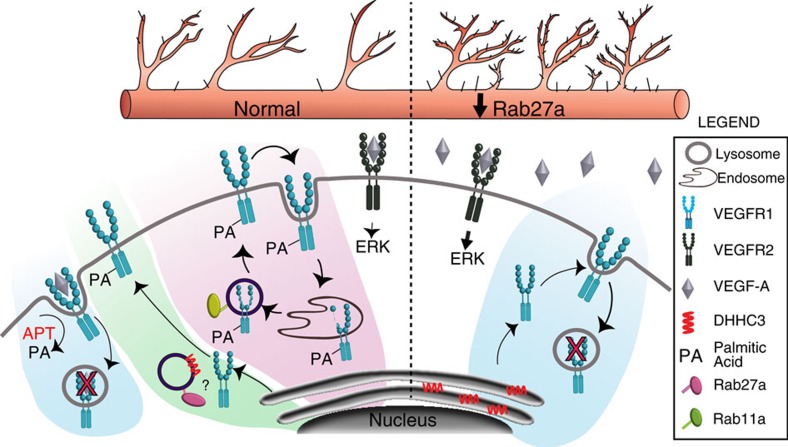
Model for regulation of mVEGFR1 stability in endothelial cells. mVEGFR1 is normally (left) highly stable and slowly recycled to and from the surface via Rab11a, requiring Rab27a and DHHC3 for palmitoylation; reduced Rab27a levels (right) destabilize mVEGFR1 via reduced palmitoylation, leading to increased signalling through VEGFR2.

VEGFR1 regulates VEGF-A signalling amplitude via its decoy function to control blood vessel growth and morphogenesis. Unlike most receptors whose primary function is signalling, membrane-localized VEGFR1 (mVEGFR1) is highly stable, and its trafficking and turnover are exquisitely regulated via post-translational palmitoylation. Disruption of this regulatory axis affects angiogenesis *in vitro* and *in vivo*. We show that mVEGFR1 palmitoylation regulates a switch from a stable, constitutively recycling mode to a degradative route that is predicted to remove ligands from the system. mVEGFR1 stability and function requires proper trafficking of palmitoylation enzymes, and Rab27a uniquely regulates angiogenesis via effects on mVEGFR1 palmitoylation (Fig. 9). This new paradigm shows that trafficking-dependent post-translational modifications affecting decoy receptor stability regulate biological outputs, and impact the fine-tuning of vascular processes in unexpected ways.

Protein half-lives vary by several orders of magnitude^58^. Short-lived proteins are usually phosphorylation targets and signal, while longer-lived proteins often function as ‘housekeeping' proteins^59^. The half-lives of VEGFR2 and VEGFR3 absent ligand are 70 min and 4 h, respectively^36^,^60^, consistent with their primary function as signalling receptors. In contrast, mVEGFR1 has a half-life greater than 28 h absent ligand. This stable pool of mVEGFR1 constitutively internalizes and likely recycles, allowing endothelial cells to rapidly modulate VEGF-A signalling cell-autonomously, without *de novo* protein synthesis. Other decoy receptors, such as the atypical chemokine receptor D6, have an extended half-life and also constitutively recycle^61^,^62^. mVEGFR1 trafficking and stability depends on Rab11a, a small GTPase associated with the ‘slow' recycling of proteins between endosomes and the cell surface, similar to D6 (refs 63, 64). Moreover, decoy receptors often traffic to lysosomes upon ligand-binding, where ligand is released and degraded while the receptor recycles, or the entire complex is degraded^63^. These findings suggest that slow recycling and extended stability are general features of negative modulators of signalling, or molecular rheostats.

The reversibility of palmitoylation imparts versatility to protein trafficking and stability. Insulin dynamically alters the endothelial palmitoyl-proteome to influence angiogenesis *in vitro*^30^. We show that mVEGFR1 stability is reversibly regulated by palmitoylation, with unbound receptor palmitoylated and highly stable, while ligand-bound receptor is destabilized in a palmitoylation-dependent manner. VEGF-A binding to mVEGFR1 is predicted to induce conformational changes^65^, and these changes may induce depalmitoylation and mVEGFR1 trafficking to the lysosome. Our finding that the stability and function of mVEGFR1 depends on palmitoylation may represent a general mechanism for stabilizing decoy receptors.

We identify a novel role for Rab27a in angiogenesis upstream of mVEGFR1. Rab27a regulates mVEGFR1 palmitoylation and stability, likely via its trafficking functions^50^,^66^. Consistent with Rab27a affecting mVEGFR1 levels, Rab27a knockdown caused mis-trafficking of the palmitoylation enzyme DHHC3, reduced baseline palmitoylation and destabilized mVEGFR1, and elevated VEGFR2 signalling *in vitro* and *in vivo*. Vascular sprouting in 3D assays and filopodia formation *in vivo* were increased by loss or reduction of Rab27a, consistent with mis-regulation of mVEGFR1 palmitoylation. Interestingly, mice homozygous for *ashen* or lacking the cytoplasmic domain of mFLT-1 that is presumably palmitoylated are viable^20^,^55^, suggesting compensation mechanisms for destabilized mFLT-1 *in vivo*. However, the reduced FLT-1 levels and vascular phenotype of *ash/ash* mutant vessels are accompanied by increased VEGF-A signalling, supporting that Rab27a regulates blood vessel formation through effects on FLT-1 stability *in vivo*.

This new angiogenic regulatory axis influences mVEGFR1 stability and trafficking, and affects the responses of developing vessels to environmental signals. Our work links palmitoylation to VEGF-A signalling for the first time, and shows that post-translational regulation of decoy receptors affects biological outputs. Moreover, the likely mechanism of mVEGFR1 palmitoylation, via an enzyme that is trafficked and regulated by Rab27a, suggests novel ways to manipulate and fine-tune blood vessel morphogenesis.

## Methods

### Cell culture

No cell lines used in this study are found in the International Cell Line Authentication Committee ICLAC commonly mis-identified cell line database. HUVEC (Lonza, #2519A) were cultured in EBM-2 medium supplemented with the bullet kit (EGM-2) (Lonza) and used at passages 3–5. Normal human lung fibroblasts (NHLF) (Lonza, #CC-2512) were maintained in DMEM supplemented with 10% newborn calf serum and used at passages 4–6. For gene knockdown in HUVEC, approximately 1 × 10^6^ cells were suspended in 100 μl nucleofector solution (Lonza) before adding 50–150 pmol of siRNA (Silencer Select Locked Nucleic Acids, Supplementary Table 3) and electroporating using the A-034 program of the Amaxa Nucleofector system. For ligand stimulation, HUVEC were starved in EBM-2 with 0.1% serum for 3 h, then 50 ng ml^−1^ human PLGF, VEGF-A or VEGF-B (Peprotech) was added. Recombinant human VEGFR1-FC (R&D, # 321-FL-050) was polymerized in fibrin gels (25 ng ml^−1^) for 3D sprouting assays. HUVEC and NHLF were certified mycoplasma-free by the UNC Tissue Culture Facility.

### Antibodies inhibitors and siRNAs

See Supplementary Tables 1–3 for antibodies, inhibitors and siRNAs used for this study. HA-tagged DHHC3 was a kind gift of Dr William Sessa (Yale University).

### Mice

All experiments involving animals were performed with approval of the University of North Carolina, Chapel Hill Institutional Animal Care and Use Committee. Ashen mice (strain C3H/HeSn-Rab27a^ash^/J) were purchased from Jackson Laboratories (# 000120), bred in-house and used for experiments from P5–P8.5. Male and female pups were used in equal proportions.

### Retina dissection

Mouse retinas were processed as described^67^. Briefly, mice were anaesthetized for 15 min with isoflurane before being killed. Eyes were collected, fixed in 4% paraformaldehyde (PFA) for 1 h, washed twice with PBS and then the retinas dissected and stored in PBS at 4 °C. Retinal lysates were generated by homogenizing retinas using a microcentrifuge-tube pestle in the presence of 200 μl RIPA with 2 × protease/phosphatase inhibitor cocktail (Cell Signaling Technology). Ground tissue was kept on ice and vortexed for 15 s every 5 min for a total of 30 min. Lysates were centrifuged at 11,000*g* for 20 min and supernatant transferred to a fresh tube. Total protein was measured using the DC protein assay (Bio-Rad), and reduced and denatured in Laemmli Buffer with 50 mM DTT at 95 °C for 20 min.

### Aortic ring assay

The aortic ring assay was carried out as described^53^. Briefly, 6-week-old male mice (C3H/HeSn) were anaesthetized with isofluorane for 5 min before being killed. The descending aorta was isolated, cleared of adventitia and placed in serum-free Opti-MEM (Gibco) with 1 × antibiotic/antimycotic (Gibco, 100 × stock solution). Aortas were sectioned into ∼25 rings of 0.5 mm thickness and placed in fresh serum-free Opti-MEM with 1 × antibiotic/antimycotic for 1 h at 37 °C. Rab27a-specific siRNA or non-targeting control were diluted to 100 nM in 480 μl Opti-mem medium containing 20 μl Lipofectamine RNAi MAX (Invitrogen) and added to 4 ml Opti-mem and ∼30 rings for overnight incubation at 37 °C. Rings were washed in PBS, and embedded in 2.5 mg ml^−1^ fibrin supplemented with 37.5 μl ml^−1^ of 1 mg ml^−1^ aprotinin per ml of fibrinogen. EBM-2 medium supplemented with 0.2% FBS and 25 ng ml^−1^ VEGF-A was added to each well. Medium was changed on day 4, then every other day.

### Sprouting angiogenesis assay

The sprouting angiogenesis assay was performed essentially as described^37^,^68^. Briefly, 5 × 106 HUVEC were trypsinized, mixed with ∼2,000 pre-washed microcarrier beads (GE Healthcare) in 2 ml EGM-2, incubated for 4 h at 37 °C with gentle flicking every 20 min, then placed in EGM-2 overnight at 37 °C. On day 2, beads were transferred to 15 ml tubes, allowed to settle and medium was aspirated. After washing thrice in PBS, beads were suspended in 2 mg ml^−1^ fibrinogen solution (90% clottable) supplemented with 37.5 μl of 1 mg ml^−1^ aprotinin per ml of fibrinogen. Five hundred microlitres of solution was added to each well of a glass-bottom 24-well plate containing 7 μl bovine thrombin (50 units per ml). The solution was gently titrated three times, a fibrin clot was formed for 3 h at 37 °C, then NHLF were overlaid (∼25,000 cells per well) in 1 ml EGM-2. The medium was changed 1 day later, then every other day.

### Immunoblot

Immunoblot was performed as previously described^69^. Briefly, cells were lysed in RIPA buffer containing protease/phosphatase inhibitor (Cell Signaling), and approximately 20–50 μg of protein were separated by SDS–PAGE and transferred to PVDF membranes. Membranes were blocked for 30 min in 5% milk in PBS supplemented with 0.1% Tween-20 (PBS-T), and incubated at 4 °C with primary antibodies in 1% non-fat milk in PBS-T overnight (Supplementary Table 1). Membranes were washed thrice in PBS-T (PBS+0.1% Tween-20) before adding HRP-conjugated secondary antibodies for 1 h at RT. Secondary antibody was removed and membranes washed four times in PBS-T before addition of Luminata Forte (Millipore). For subcellular fractionations, sequential lysing was carried using the Cell Fractionation Kit (Cell Signaling #9038), according to the manufacturer's protocol, before running lysates on SDS–PAGE and blotting.

### HUVEC immunofluorescence

Coverslips were prepared by washing in 100% ethanol, followed by passing the coverslip through a flame and then storing in 100% ethanol. Before seeding cells, coverslips were washed in PBS and overlaid with 0.1% gelatin for 15 min. Before imaging, cells were washed twice with PBS, fixed in 4% PFA for 10 min at RT and permeabilized with 0.5% Triton X-100 for 5 min. Blocking was performed for 30 min at RT in a solution in PBS supplemented with 5% goat serum, 2% BSA and 0.1% Tween-20 (staining solution) before addition of antibody (Supplementary Table 1) overnight at 4 °C. The next day, cells were washed five times with PBS-T and incubated with Alexa-fluor-conjugated anti-species secondary (1:1,000) for 1 h at RT. Cells were then washed five times with PBS-T and incubated with Alexa-fluor-conjugated phallodin (1:500) and DRAQ7 (1:1,000) in PBS for 30 min to visualize F-actin and nuclei, respectively. Coverslips were mounted to slides using Fluorogel inTris buffer (Electron Microscopy Sciences) and sealed with nail polish.

### HUVEC sprout immunofluorescence

Fibroblasts were removed using 0.25% trypsin for ∼1 min, and fibrin gels washed thrice with PBS. Gels were fixed with 4% PFA for 30 min, then permeabilized using 0.5% Triton X-100 in PBS for 1 h at RT. Wells were washed in PBS and fixed with 4% PFA in PBS for 30 min. Sprouts were permeabilized with 0.5% Triton X-100, washed thrice with PBS, blocked in staining solution for 3 h at RT (see previous section), then incubated with primary antibody at 4 °C for 48 h (Supplementary Table 1). Gels were washed for 24 h with 0.5% Tween-20 and incubated with Alexa fluor-conjugated secondary for 4 h at RT. Gels were washed 5 × 20 min with 0.5% Tween-20 and incubated overnight at 4 °C with phalloidin (1:200) or DRAQ7 (1:1,000) in PBS.

### Aortic ring immunofluorescence

Staining of mouse aortic rings was performed essentially as described^53^, and as indicated above, with a few exceptions. Rings were fixed with 4% PFA for 1 h at RT, then permeabilized in 1% Triton X-100 for 45 min at RT. Rings were blocked in 2% BSA/5% goat serum/0.5% Tween-20 overnight at 4 °C, then incubated with primary antibody/secondary antibody as described above. Before imaging, rings were incubated with Alexa-fluor 488 conjugated isolectin (1:100) for 1 h at RT.

### Retina immunofluorescence

Retinas were dissected, cleaned and dehydrated in 100% ethanol for 30 min followed by permeabilization with 1% Triton X-100 for 30 min at RT. Retinas were blocked in retina staining solution (5% goat serum, 1% BSA, 1% Triton X-100) for 3 h at RT, followed by overnight incubation with primary antibody at 4 °C. Retinas were washed four times with 0.5% triton X-100, followed by incubation with Alexa fluor-conjugated secondary antibody for 3 h at RT. Retinas were washed four times, counterstained with isolectin (1:100) at 4 °C overnight, and mounted using fluoro-gel.

### Internalization assays

Internalization assays were performed essentially as described^38^, with slight modification. For VEGFR1 internalization, HUVEC were washed five times with pre-chilled PBS supplemented with Ca+2 and Mg+2 (PBS+) on ice at 4 °C. Following washing, HUVEC were incubated with ice-cold blocking/internalization solution EBM-2 supplemented with 0.5% w/v BSA for 30 min on ice at 4 °C to prevent internalization. Alexa-fluor-488 conjugated primary antibody against VEGFR1 (Abcam Cat # 195253) or IgG isotype control (Cell Signaling Technologies Cat t# 4340) was added (1:50) to pre-chilled blocking solution incubated with HUVEC for 2 h at 4 °C to label cell surface proteins. After 2 h, cells were washed five times in ice cold PBS+ before adding pre-warmed (37 °C) internalization medium to each plate and incubating at 37 °C for 45 min to activate endocytosis. When striping of the cell surface was performed, HUVEC were incubated with 0.5 NaCl/0.2 M acetic acid for 4 min after internalization but before fixation to remove antibody from cell surface receptors.

### Cell surface protein isolation

Biotinylation and isolation of cell surface proteins was performed according to the manufacturer's protocol (Cell Surface Protein Isolation Kit, Pierce cat# 89881). Briefly, control and 2-BH-treated HUVEC (see Supplementary Table 2) were washed twice in 10 ml of ice-cold PBS to stop internalization and incubated with EZ-link Sulfo-NHS-SS-Biotin at 4 °C for 30 min with constant rocking. HUVEC were washed twice with ice-cold PBS and removed from the plate by gently scraping. After centrifugation at 500*g* for 3 min, cells were lysed using provided lysis buffer supplemented with 1 × protease/phosphatase inhibitors (Cell Signaling Technologies). Biotinylated proteins were captured using NeutrAvidin Agarose slurry-containing spin columns. Flow-through was saved for analysis, and the column was eluted with 1 M DTT. Total protein from the eluate (labelled proteins) and flow through (unlabeled proteins) were analysed by immunoblot.

### Acyl biotin exchange assay

Acyl Biotin Exchange was performed essentially as described^43^. Semi-confluent HUVEC were pooled, lysed on ice in 1% IGEPAL dissolved in (50 mM Tris-HCL pH 7.5, 150 mM NaCL, 10% glycerol with PMSF and protease/phosphatase inhibitor) for 30 min in the presence of 10 mM N-ethylmaleimide (NEM) to block free cysteines. Lysates were cleared by centrifugation (16,000*g* for 30 min). Five micrograms of αVEGFR1 antibody and fresh NEM was added to the supernatant and incubated overnight at 4 °C. The following day, 100 μl protein A/G agarose beads were added to the solution and rotated for 2 h at 4 °C to precipitate VEGFR1. After centrifugation (0.5*g*), beads were resuspended in 600 μl lysis buffer containing 10 mM NEM and split to input, minus hydroxylamine (−HAM) and plus (+HAM) conditions before washing with lysis buffer containing 0.1% SDS. Beads were resuspended in lysis buffer+1 M HAM (or molar equivalent of NaCl for negative control) and rotated for 2 h at 37 °C. Beads were centrifuged and washed thrice in lysis buffer with 10 mM NEM. Biotin-BMCC was freshly prepared in DMSO and added to a final concentration of 2.0 μM in lysis buffer, and samples were rotated for 1 h at 4 °C. Beads were washed thrice in lysis buffer containing protease/phosphate inhibitors, and VEGFR1 was eluted from beads by addition of Laemmli sample buffer with 100 mM DTT at 90 °C for 20 min. Input, −HAM and +HAM conditions were analysed by immunoblot using streptavidin-HRP, then stripped and re-probed with VEGFR1 antibody.

### Acyl resin-assisted capture (Acyl-RAC) assay

Acyl resin-assisted capture was performed as described^42^. For 2-BH experiments, semi-confluent HUVEC were treated with 5 μM 2-BH or EtOH for 12 h to block palmitoylation. For Rab27a knockdown experiments, HUVEC were transfected with 100 pmol of Rab27a-specific or non-targeting control siRNA (see cell culture section for details) 2 days before the experiment. Non-targeting controls and siRab27a-transfected HUVEC were incubated with 10 μg ml^−1^ chloroquine for 2 h at 37 °C. After experimental manipulations, HUVEC were mechanically scraped from the dish in ice cold PBS and pelleted by centrifugation at 200*g* for 10 min at 4 °C. PBS was removed and the pellets suspended in 200 μl lysis buffer (25 mM HEPES, 25 mM NaCl, 1 mM EDTA, 0.5% Triton X-100, pH 7.5) with protease/phosphatase inhibitors on ice for 30 min. Whole retinas were lysed in RIPA (see above) with 2 × protease phosphatase inhibitors using a tube pestle. Lysates were centrifuged at 11,000*g* for 20 min, and the supernatants were collected for further processing. Equal amounts of protein from each sample were diluted to 2 mg ml^−1^ in blocking buffer (100 mM HEPES, 1.0 mM EDTA, 2.5% SDS, pH 7.5) containing 0.1% methanethiosulfonate and incubated at 40 °C for 12 min, with vortexing every 2 min, to irreversibly block free cysteines. Proteins were precipitated by adding 3-volumes of 100% ice cold acetone and incubating at −20 °C for 30 min, then centrifuging at 5,000*g* for 10 min. Protein pellets were washed thrice with 70% acetone, dried and re-suspended in 300 μl binding buffer (100 mM HEPES, 1.0 mM EDTA, 1% SDS, pH 7.5) containing protease/phosphatase inhibitors. Twenty microlitres of solution was set aside for input control. Fifty microlitres pre-washed thiopropyl sepharose 6B (Sigma cat # T8387) was added to each sample, followed by addition of 40 μl of 2 M HAM (pH 7.5) (+HAM condition) or 40 μl of 2 M NaCl (−HAM condition). Samples were rotated at 37 °C for 2 h. Thiopropyl sepharose beads were washed four times in binding buffer, and captured proteins were eluted from the beads by addition of 50 mM DTT in 50 μl binding buffer for 20 min. Eluted proteins were then boiled at 90 °C for 20 min in Laemmli sample buffer (375 mM Tris-HCL pH 6.8, 9% w/v SDS, 50% glycerol, 0.03% bromophenol blue, 100 mM DTT) and analysed by immunoblot.

### Imaging

All imaging was done using an Olympus FV1200 Laser Scanning Confocal Microscope (60 × oil objective with 1.4 NA or 40 × silicone objective with 1.25 NA) and Flow View software.

### Image and statistical analysis

Priori power analysis determined a minimum sample size of 5 pups per group (alpha 0.05, effect size 20%) for 90% power when calculating differences in retinal vessel area and plexus filopodia. All quantifications were measured and analysed by a blinded researcher. For quantitative immunofluorescence image analysis, corrected total cell fluorescence was calculated for individual cells using integrated density (mean fluorescence of the area × cell area) (http://fiji.sc/). Normalized data sets were graphed and analysed using PRISM. For two-sample data sets with equal variances (control versus a single experimental condition) unpaired, two-tailed Student's *t*-test was used as reported in figure legends. For data sets with greater than two conditions and equal variances, one-way ANOVA with Tukey's *post-hoc* test was used as reported in the figure legends. *P≤0.05, **P≤0.01, ***P≤0.001, ns, not significant.

### Data availability

All data in support of the findings of this work can be found within the article and its Supplementary Information, and from the corresponding author on reasonable request.

## Additional information

**How to cite this article:** Boucher, J. M. *et al*. Dynamic alterations in decoy VEGF receptor-1 stability regulate angiogenesis. *Nat. Commun.* **8,** 15699 doi: 10.1038/ncomms15699 (2017).

**Publisher's note:** Springer Nature remains neutral with regard to jurisdictional claims in published maps and institutional affiliations.

## Supplementary Material

Supplementary Information Supplementary Figures and Supplementary Tables.
