# Supplementary material for: Dynamic alterations in decoy VEGF receptor-1 stability regulate angiogenesis
Source: Nat Commun. 2017 Jun 7;8:15699. doi: 10.1038/ncomms15699 (PMC5467243; doi:10.1038/ncomms15699)
Supplement: Supplementary Information — Supplementary Figures and Supplementary Tables. [file ncomms15699-s1.pdf]

Supplementary Figure 1

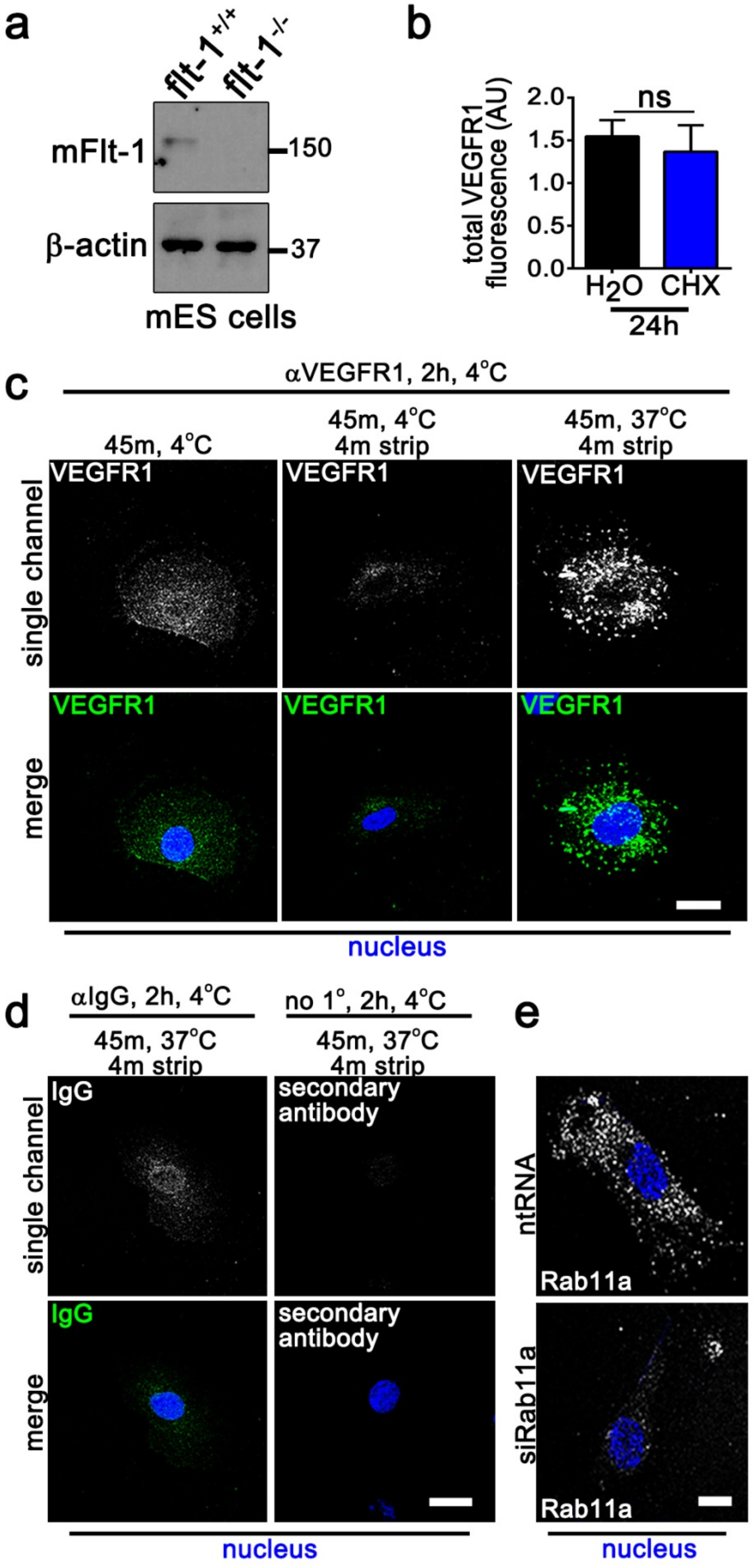

Supplementary Figure 1 (linked to Figure 1). mVEGFR1 constitutively internalizes in endothelial cells.
(a) Immunoblot of mFlt1 from undifferentiated mouse embryonic stem (ES) cells with indicated genotypes; 2 replicates.
(b) Quantification of VEGFR1 fluorescence intensity of HUVEC with indicated treatments via integrated density. (n=44/control; n=42/CHX). CHX, cycloheximide; 2 replicates. Statistics: Shown are means + 95% CI, student's t-test with Tukey's post-hoc range test; ns, not significant.
(c-d) Immunofluorescence of VEGFR1 from HUVEC internalization assay with indicated treatments. Strip: 0.2M acetic acid, 4min; 3 replicates.
(e) Rab11a immunofluorescence in HUVEC with indicated treatments. DRAQ7 (nuclei); 2 replicates. All scale bars: 20μm.

Supplementary Figure 2

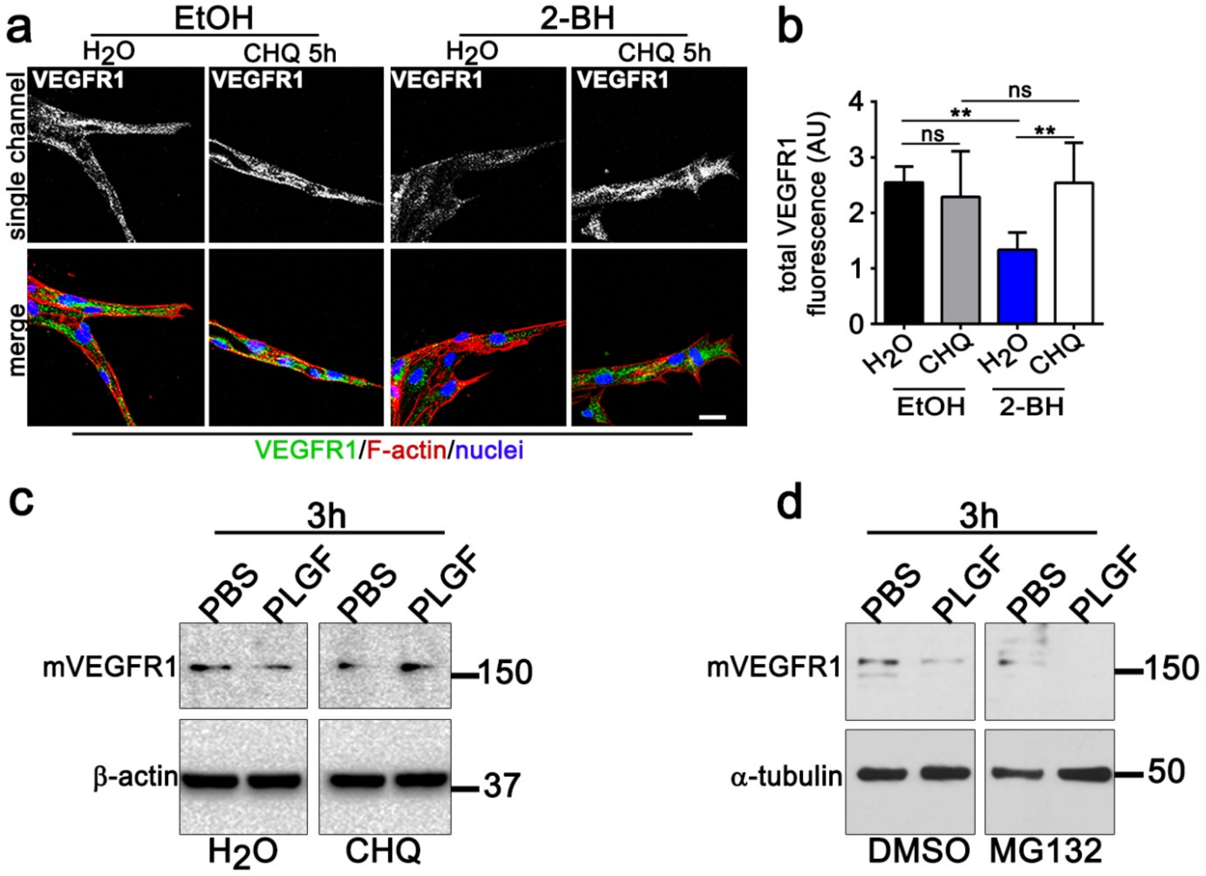

Supplementary Figure 2 (linked to Figure 2). Destabilized mVEGFR1 degrades via the lysosome.
(a-b) (a) VEGFR1 immunofluorescence of d5 HUVEC angiogenic sprouts treated with 2-BH for 12h prior to CHQ for 5h, scale bar: 25µm. (b) Quantification of VEGFR1 fluorescence via integrated density. Shown are means + 95% CI (#sprouts; EtOH/H_2O, n=6; EtOH/CHQ, n=6; 2-BH/H_2O, n=8; 2-BH/CHQ, n=5); 2-BH, 2-bromohexadecadnoic acid; CHQ, chloroquine; 2 replicates. Statistics: one-way ANOVA and pairwise comparison with post-hoc Tukey's range test. **, $p \leq 0.01$; ns, not significant.
(c-d) Immunoblot of HUVEC with indicated treatments, 3 replicates.

Supplementary Figure 3

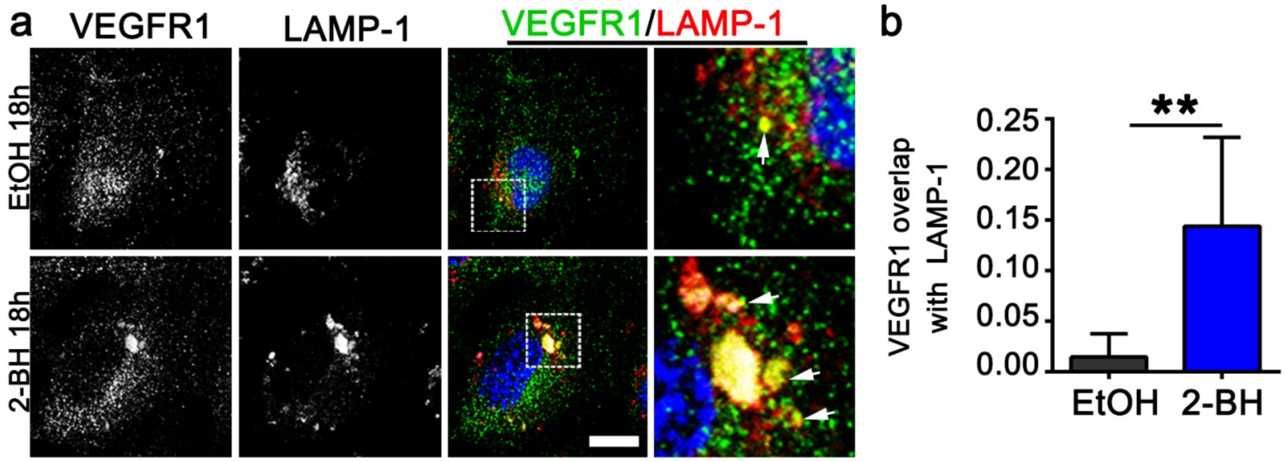

Supplementary Figure 3 (linked to Figure 3). Palmitoylation regulates mVEGFR1 localization with lysosomes.
(a-b) (a) VEGFR1 and LAMP-1 (lysosome marker) immunofluorescence of HUVEC with indicated treatments and (b) Mander's Correlation Coefficient quantification of VEGFR1 overlap with LAMP-1. (#cells: EtOH, n=38; 2-BH, n=36); 3 replicates, scale bar: 10μm.
Statistics: Shown are means +95% CI. Pairwise comparison with post-hoc Tukey's range test. **, p≤0.01.

Supplementary Figure 4

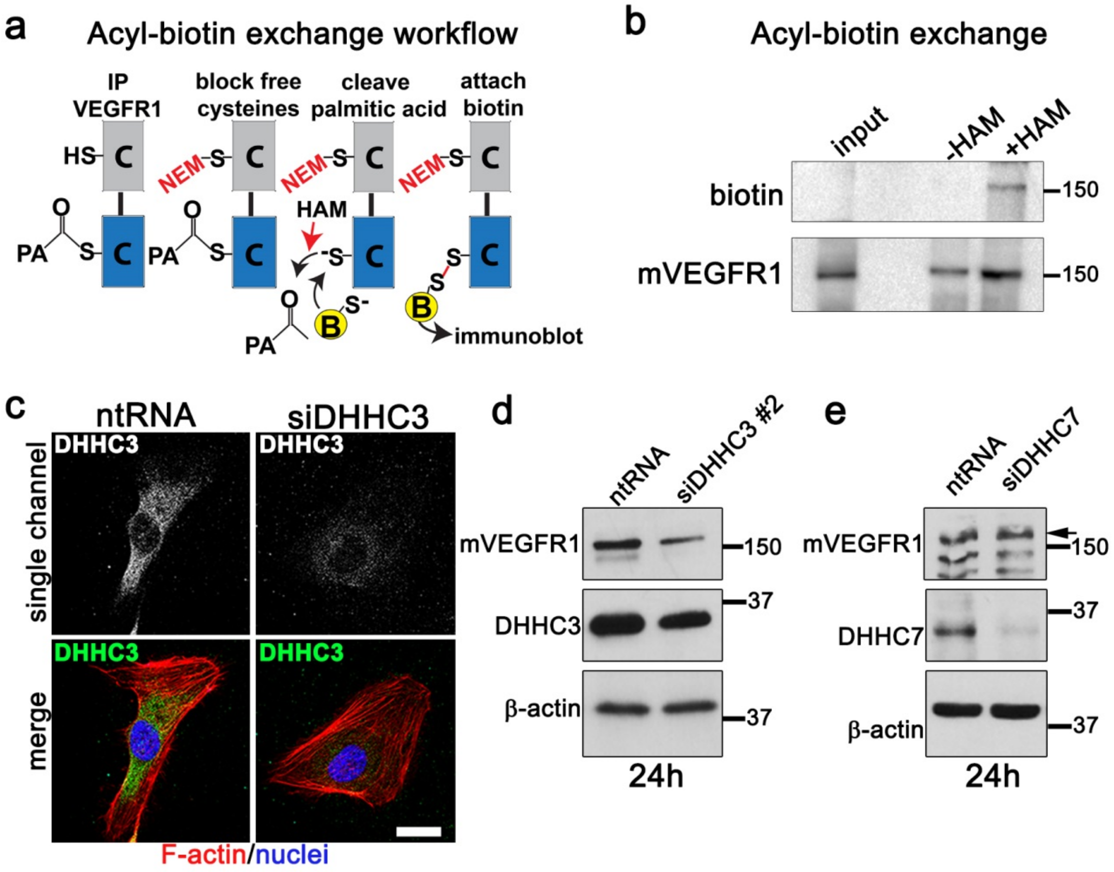

Supplementary Figure 4 (linked to Figure 4). Knockdown of the palmitoyl acetyl transferase DHHC3 reduces mVEGFR1 levels in endothelial cells.

(a) Acyl-biotin exchange workflow. B, biotin; PA, palmitic acid; NEM, N-ethylmaleimide; HAM, hydroxylamine.

(b) Acyl-biotin exchange on immunoprecipitated VEGFR1 from HUVEC with indicated treatments, then immunoblot for biotin, stripped and reprobed for VEGFR1. HAM, hydroxylamine; 2 replicates.

(c) DHHC3 immunofluorescence of HUVEC with indicated treatments for 24h; 2 replicates, scale bar: 20µm.

(d-e) Immunoblot for mVEGFR1 in HUVEC with 24h knockdown of DHHC3 (d) or DHHC7 (e); 3 replicates.

Supplementary Figure 5

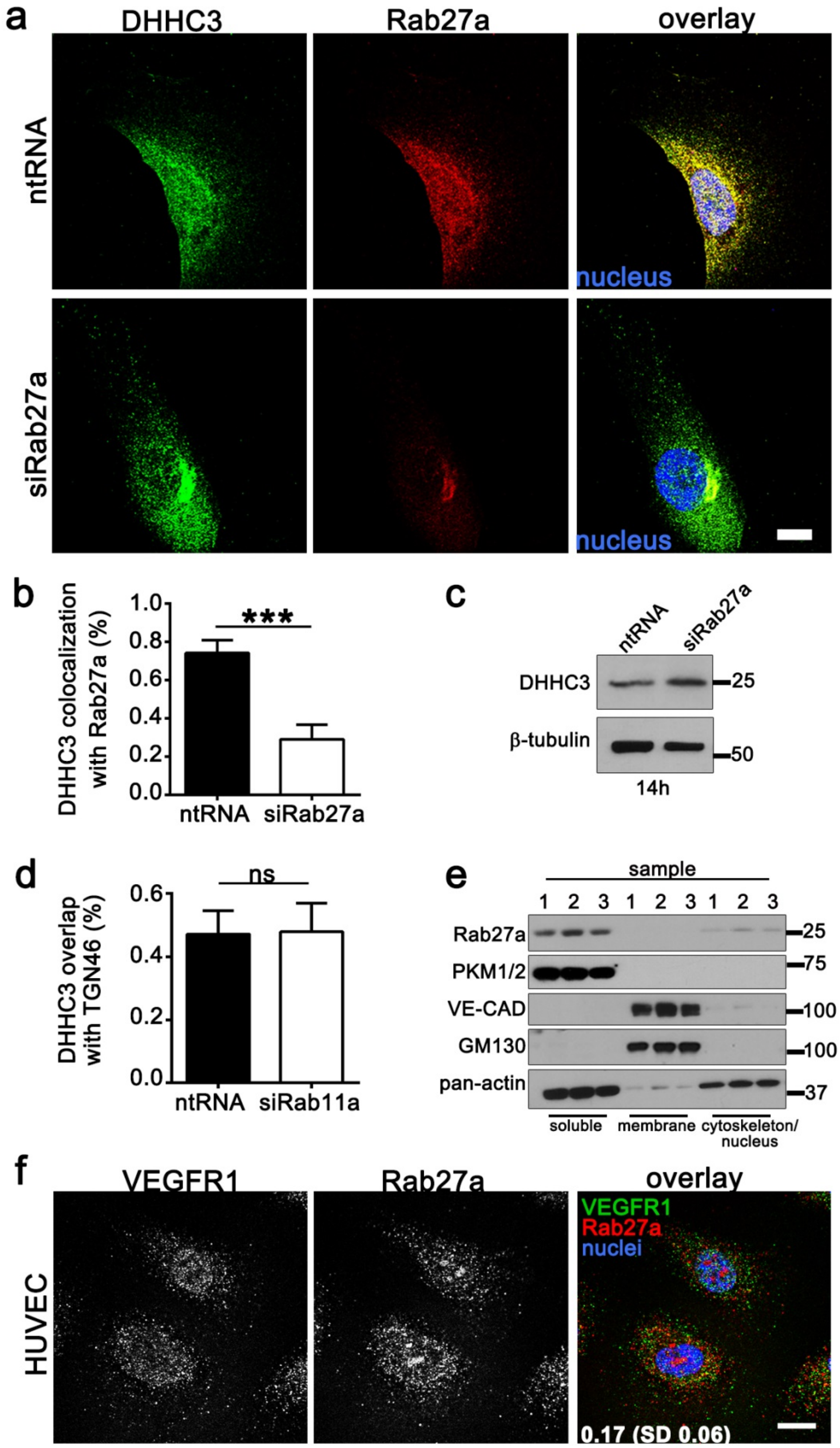

Supplementary Figure 5 (linked to Figure 5). Rab27a and DHHC3 but not VEGFR1 colocalize in endothelial cells.

(a-b) (a) Immunofluorescence of DHHC3 and Rab27a in HUVEC with indicated treatments 24h post-knockdown, scale bar: 10µm. (b) Mander's Colocalization Coeffcient quantification of overlap. Shown are means +95% CI. (#cells: ntRNA, n=31; siRab27a, n=20); 2 replicates.

(c) Immunoblot of DHHC3 in HUVEC with indicated treatments.

(d) Mander's Colocalization Coefficient quantification of DHHC3 and TGN46 (Golgi) overlap in HUVEC with indicated treatments. Shown are means + 95% CI. (#cells: ntRNA, n=19; siRab11a, n=27); 2 replicates.

(e) Subcellular fractionation and immunoblot of HUVEC from 3 different lots. Pyruvate kinase1/2 (PKM1/2), soluble marker; vascular-endothelial cadherin (VE-CAD), membrane and cytoskeletal marker; cis-Golgi Marker 130 (GM130), membrane marker; pan actin, soluble (G-actin) and cytoskeletal (F-actin) marker. 2 replicates.

(f) Immunofluorescence of VEGFR1 and Rab27a overlap quantified with Mander's Correlation Coefficient, +/- SD, scale bar 15µm.

Statistics: Student's t-test with post-hoc Tukey's range test. ***, $p \leq 0.001$; ns, not significant.

Supplementary Figure 6

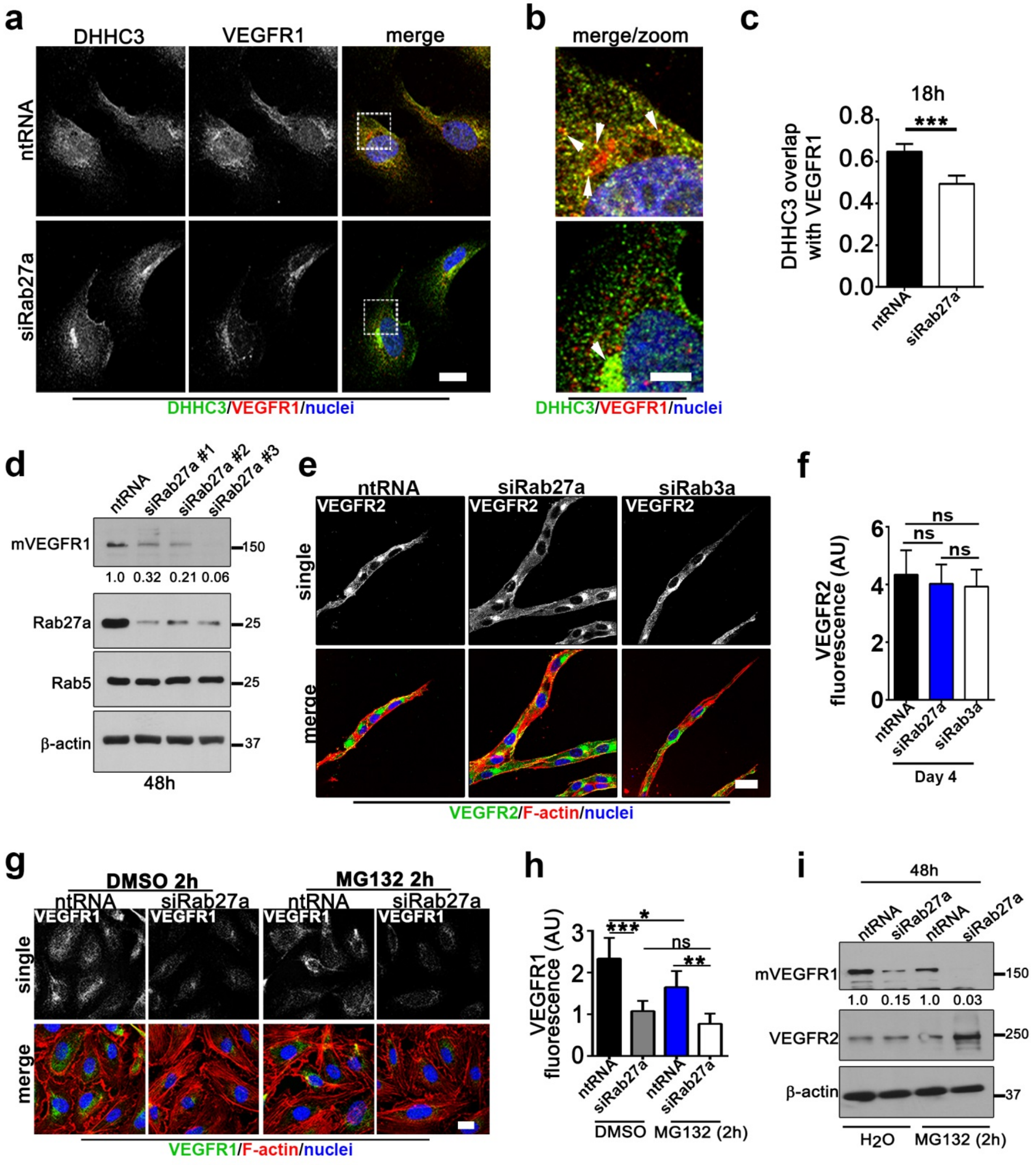

Supplementary Figure 6 (linked to Figure 5). Rab27a regulates steady-state mVEGFR1 levels.
(a-c) (a-b) Immunofluorescence of DHHC3 and VEGFR1 in HUVEC with indicated treatments, scale bars: 10μm (a) and 5μm (b). (c) Mander's Colocalization Coefficient quantification of fluorescence. (#cells: ntRNA, n=33, siRab27a, n=40); 3 replicates.
(d) Immunoblot of HUVEC with indicated treatments. Values are relative mVEGR1 levels.
(e-f) (e) VEGFR2 immunofluorescence and (f) quantification of fluorescence via integrated density of d4 HUVEC angiogenic sprouts with indicated treatments. Scale bar: 20μm, (#sprouts: ntRNA, n=10; siRab27a, n=12; siRab3a, n=7); 3 replicates.
(g-h) (g) VEGFR1 immunofluorescence of HUVEC 48h post KD and (h) quantification of fluorescence via integrated density, scale bar 15μm. (# cells: DMSO/ntRNA, n=14; MG132/ntRNA, n=18; DMSO/siRab27a, n=14; MG132/siRab27a, n=16); 2 replicates.
(i) Immunoblot of HUVEC with indicated treatments. Values are relative mVEGR1 levels; 2 replicates.
Statistics: Shown are means +95% CI. One-way ANOVA and pairwise comparison with post-hoc Tukey's range test. *, p≤0.05; **, p≤0.01; ***, p≤0.001; ns, not significant.

Supplementary Figure 7

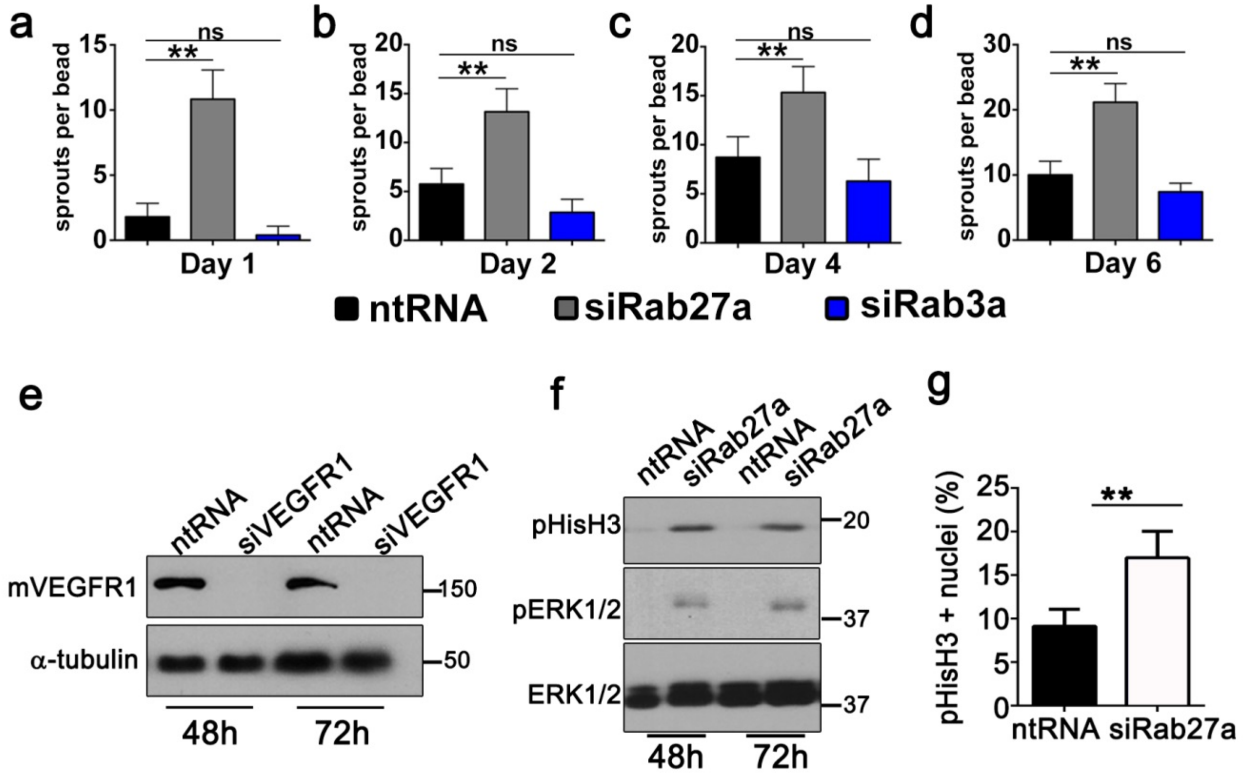

Supplementary Figure 7 (linked to Figures 6 and 7). Rab27a negatively regulates angiogenesis and increases HUVEC proliferation.
(a-d) Quantification of sprouting from HUVEC with indicated treatments on indicated days. (n=10 beads/condition/time point); 3 replicates.
(e) Immunoblot of HUVEC with indicated treatments and times; 2 replicates.
(f) Immunoblot of HUVEC with indicated treatments and times; 3 replicates.
(g) Quantification of pHisH3 (mitotic) nuclei of HUVEC 48h post-knockdown (#nuclei: ntRNA, n=682; siRab27a, n=549); 2 replicates.
Statistics: (a-d) Shown are means +95% CI. One-way ANOVA and pairwise comparison with post-hoc Tukey's range test. (g) Shown are means +SEM, Student's *t*-test. **, p≤0.01; ns, not significant.

Supplementary Figure 8

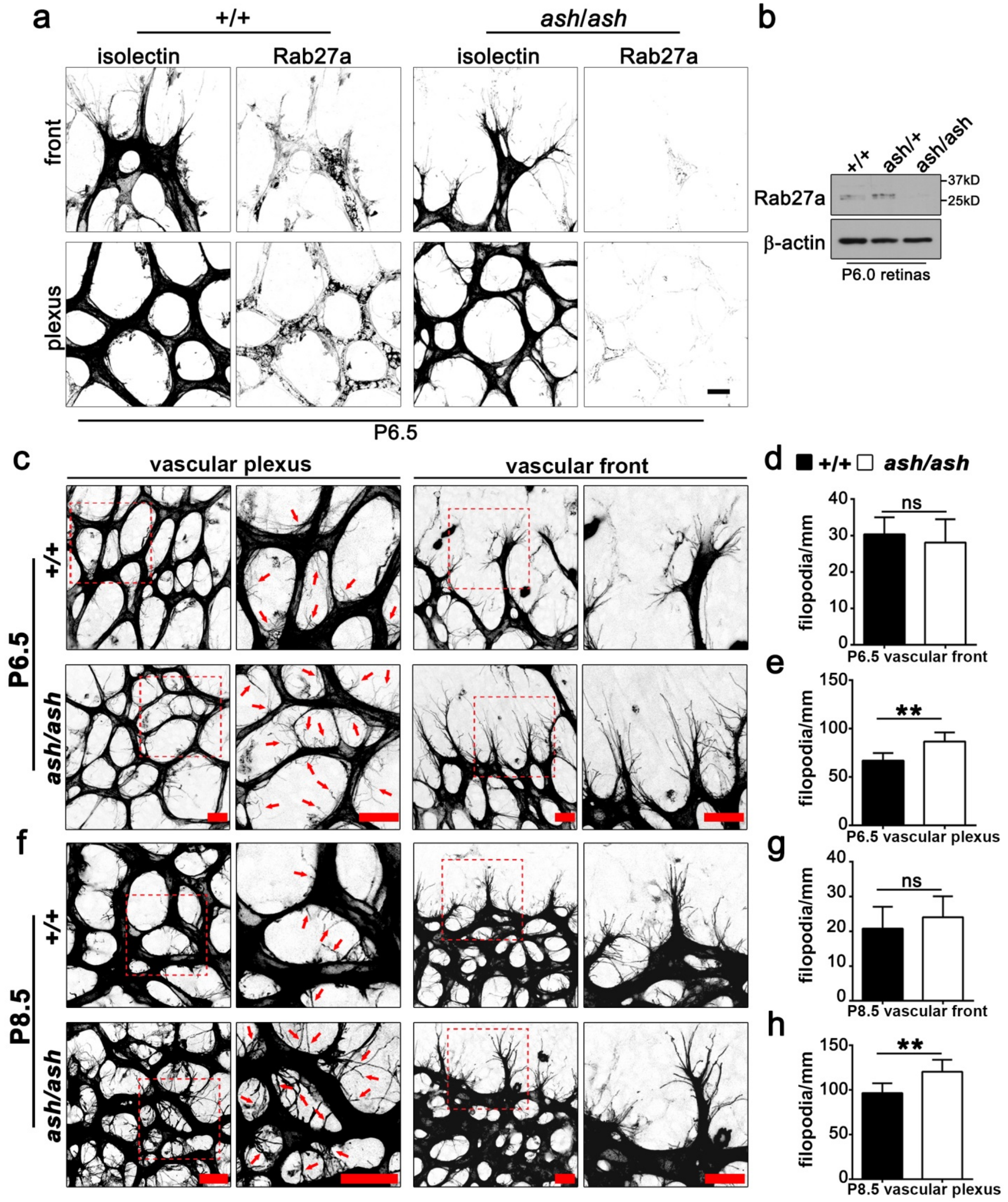

Supplementary Figure 8 (linked to Figure 8). Loss of Rab27a protein in *ash/ash* mice leads to hyperactive retinal blood vessels.
(a) Immunofluorescence of P6.5 mouse retinas for vessels (isolectin) and Rab27a of indicated genotypes; 3 replicates, scale bar: 15µm.
(b) Immunoblot of Rab27a from P6.0 retinal lysates of indicated genotypes; 4 replicates.
(c-e) (c) Immunofluorescence of P6.5 retinal vessels with indicated genotypes. Filopodia were quantified at the vascular front (d) and within the plexus (e). Red boxes, higher magnification to right. Arrows, filopodia. (#retinas: 2 independent litters: +/+, n=7*; ash*/*ash,* n=6).
(f-h) (f) Immunofluorescence of P8.5 retinal vessels with indicated genotypes. Filopodia were quantified at the vascular front (g) and within the plexus (h). Red boxes, higher magnification to right. Arrows, filopodia. (#retinas: 2 independent litters: +/+, n=6*; ash*/*ash,* n=4).
Statistics: Shown are means +95% CI. Students *t*-test. *, $p \leq 0.05$; **, $p \leq 0.01$; ***, $p \leq 0.001$; ns, not significant. Scale bars, 25µm.

Supplementary Figure 9

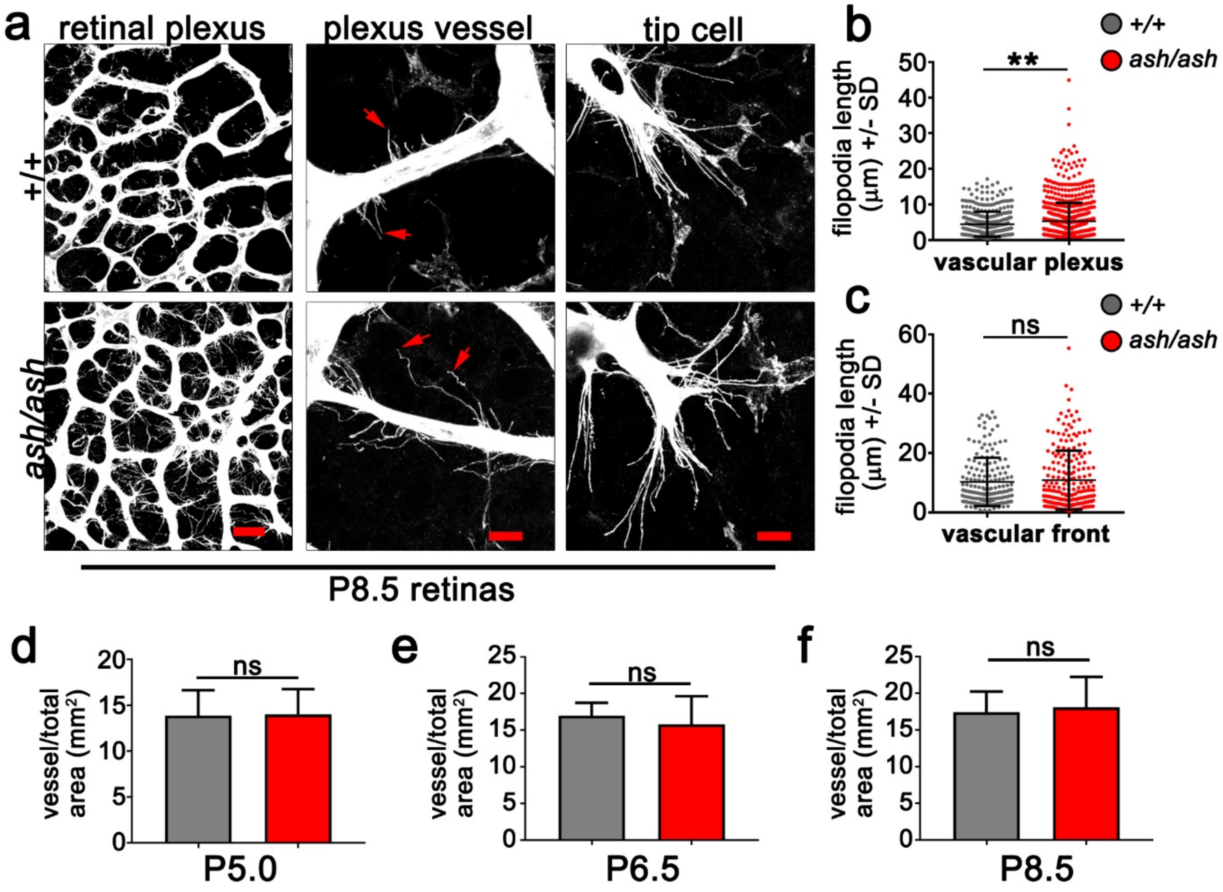

Supplementary Figure 9 (linked to Figure 8). Retinal vessels from *ash/ash* mice have longer plexus filopodia and model for regulation of mVEGFR1 stability.
(a-c) (a) Immunofluorescence of filopodia in P8.5 retinal vessels of indicated genotypes (left, low magnification plexus, scale bar, 25μm left column; middle, high magnification plexus; right, high magnification tip cells, scale bars 10μm, middle and right columns). (b-c) Quantification of filopodia length from vascular plexus (b) and vascular front (c). (#filopodia: plexus: +/+, n=396; *ash/ash*, n=827; front: +/+, n=175; *ash/ash*, n=220); 2 independent replicates.

Statistics: Shown are means +95% CI. One-way ANOVA and pairwise comparison with post-hoc Tukey's range test. **, p≤0.01; ns, not significant.

Supplementary Figure 10 (linked to all Figures). Uncropped Western blots.

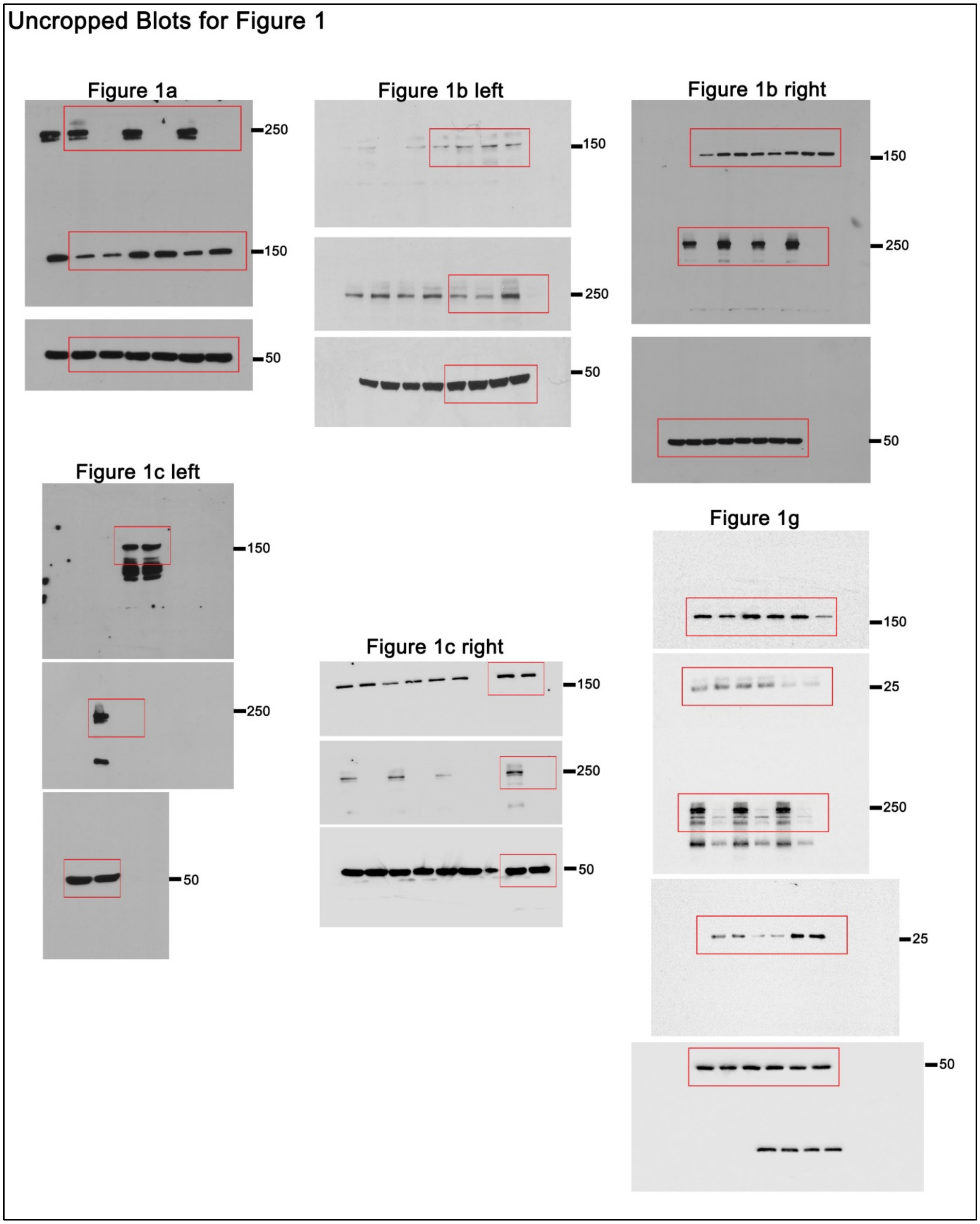

Uncropped Blots for Figure 2

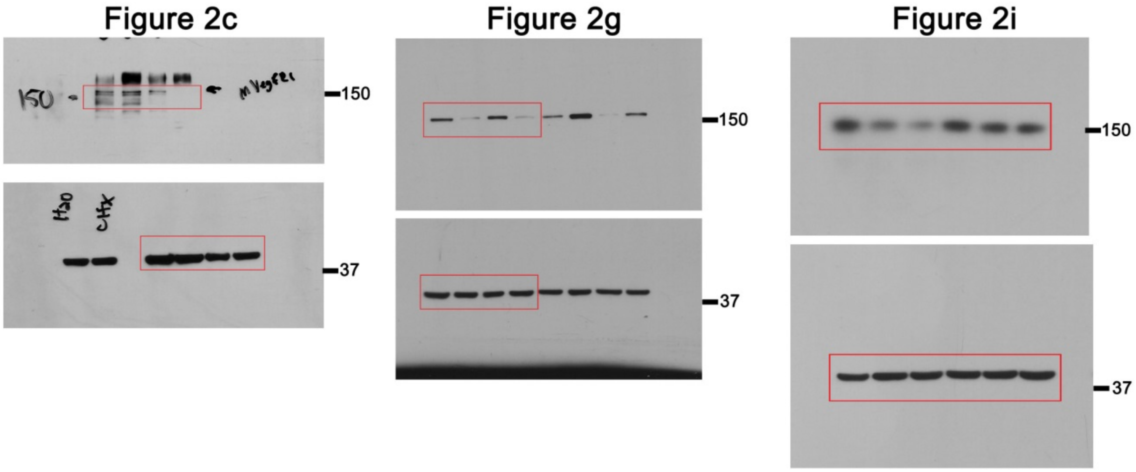

Uncropped Blots for Figure 3

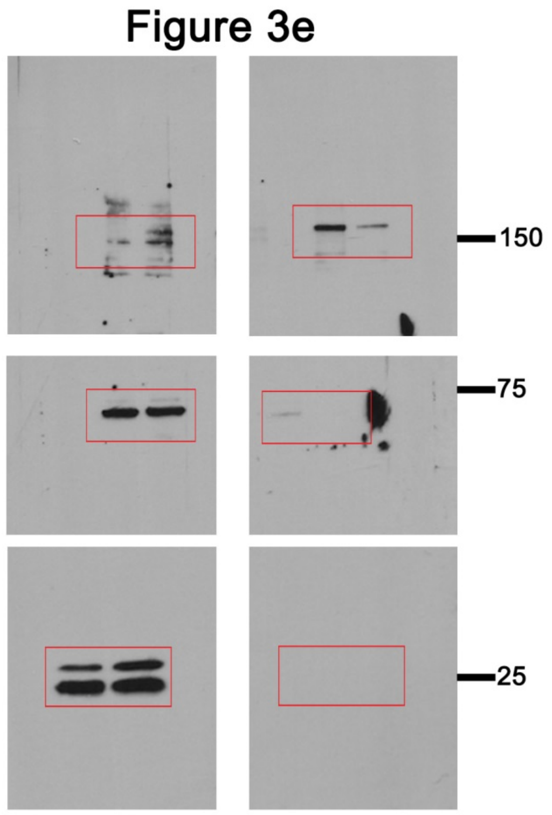

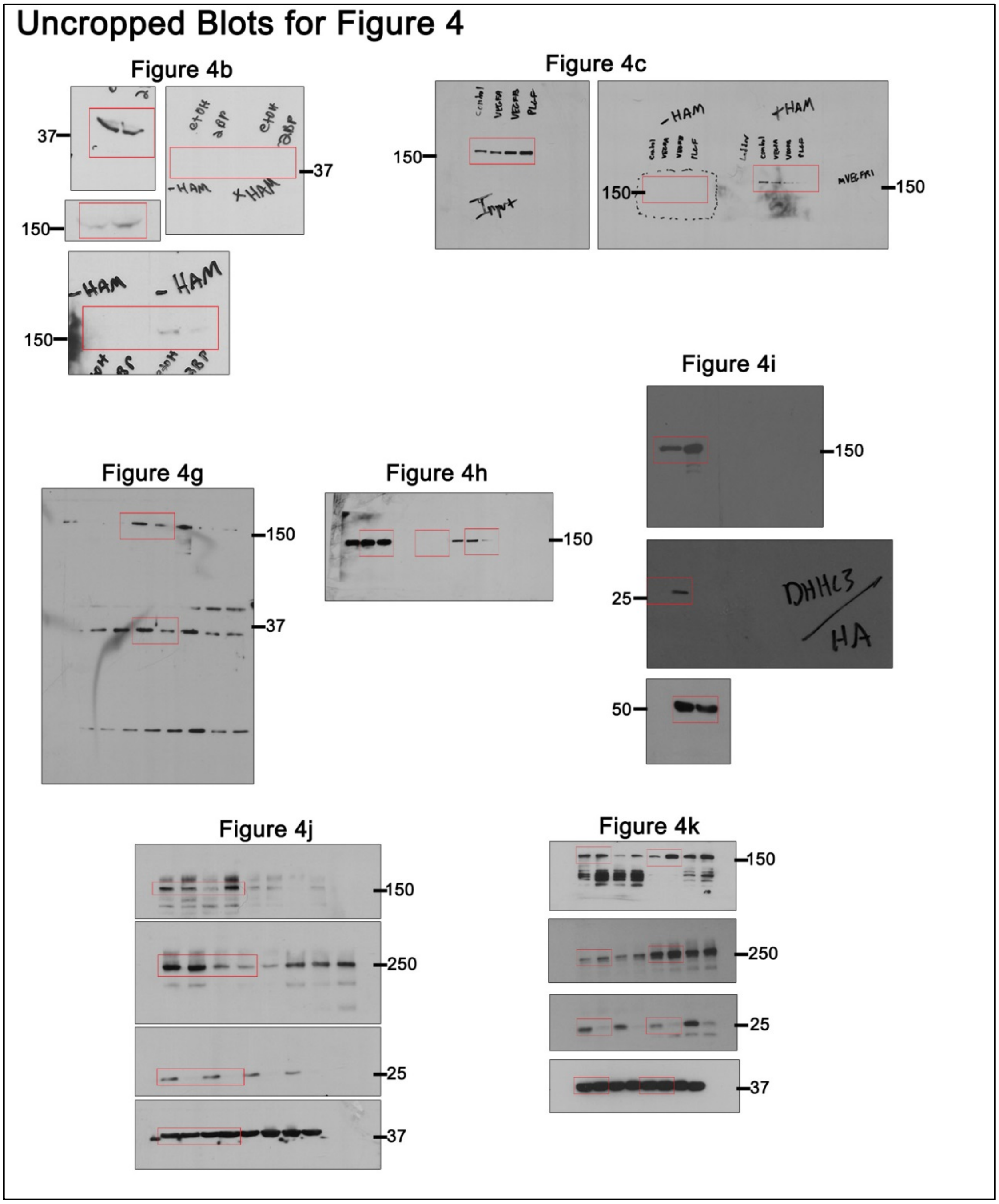
Uncropped Blots for Figure 4
Figure 4b
37
150
37
150
-HAM
-HAM
Figure 4c
150
Input
-HAM
+HAM
150
150
Figure 4i
150
25
DHHC3
HA
50
Figure 4g
150
37
Figure 4h
150
Figure 4j
150
250
25
37
Figure 4k
150
250
25
37

Uncropped Blots for Figure 5

Figure 5c

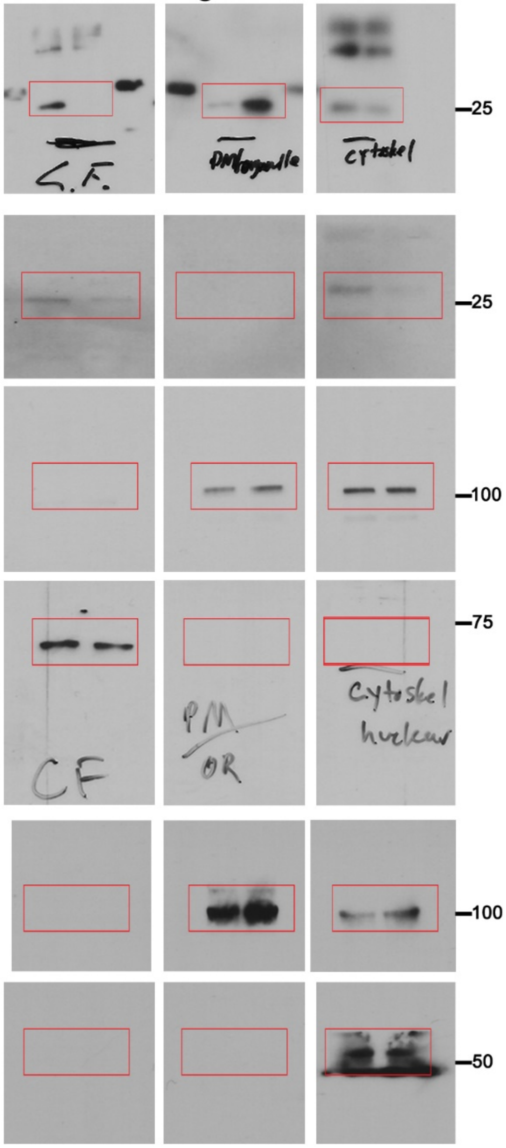

Figure 5f

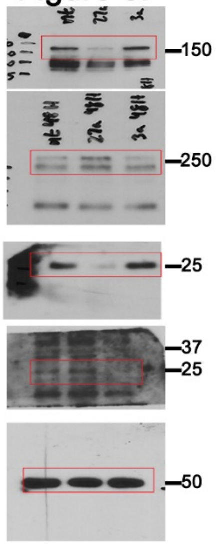

Figure 5l

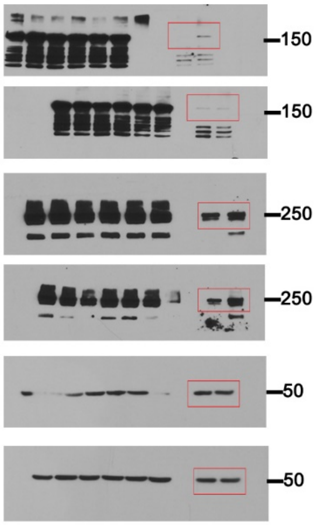

Figure 5j

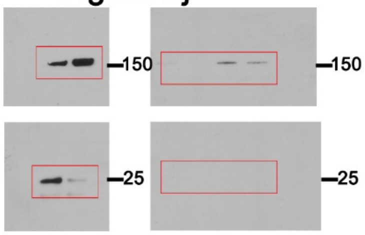

Uncropped Blots for Figure 7

Figure 7c

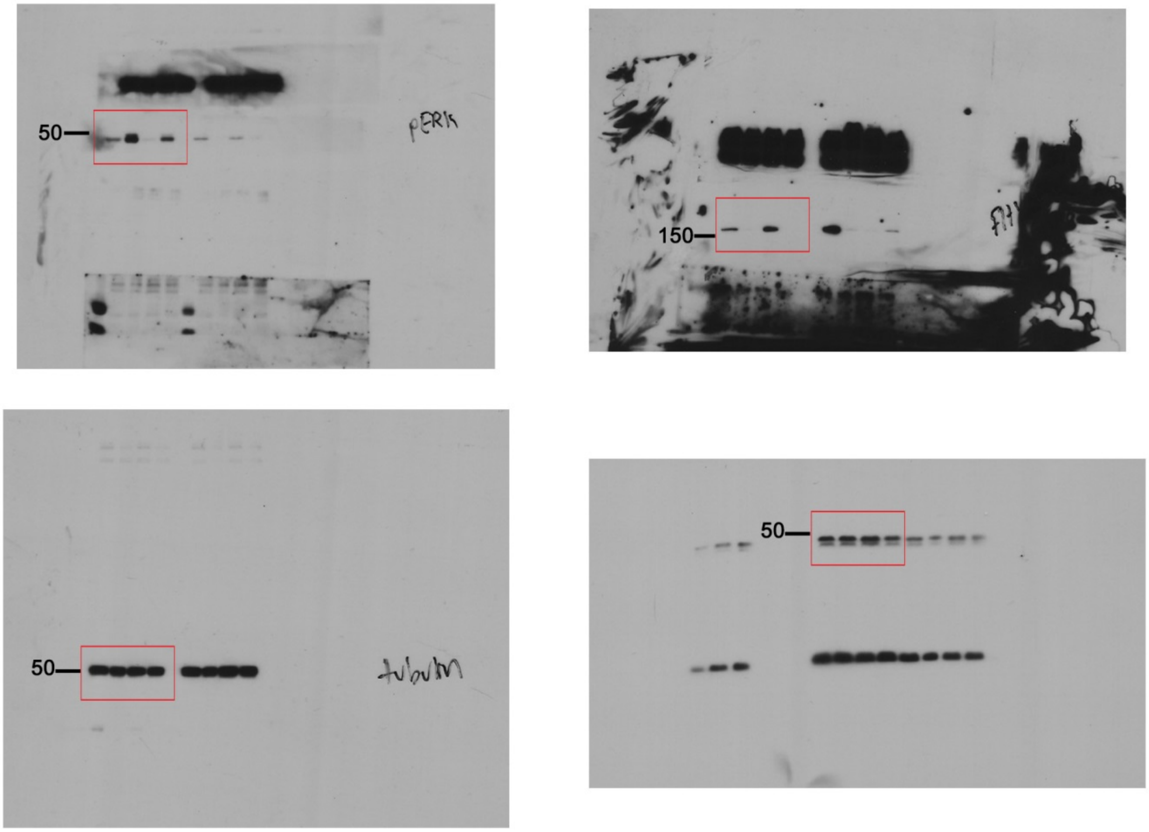

Uncropped Blots for Figure 8

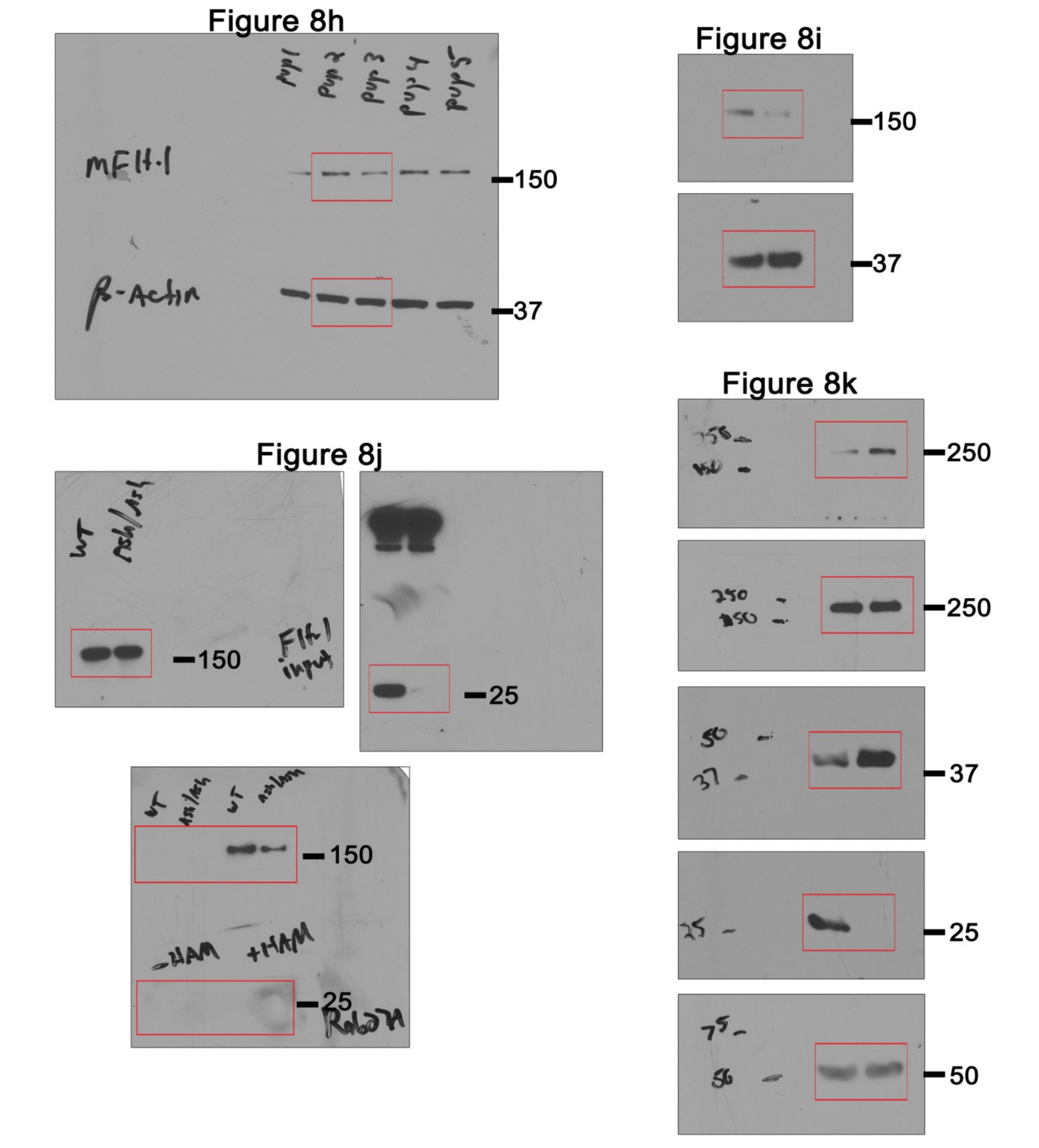

Uncropped Blots for Supplementary Figure 1

Supplementary Figure 1a

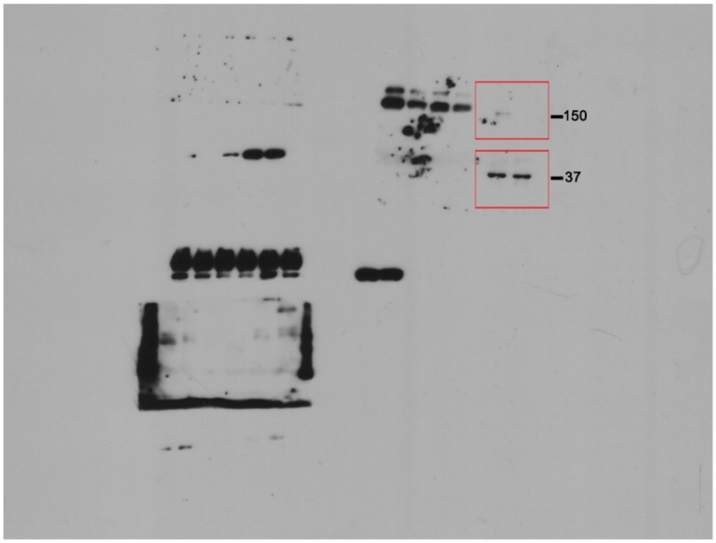

Uncropped Blots for Supplementary Figure 2

Supplementary Figure 2c

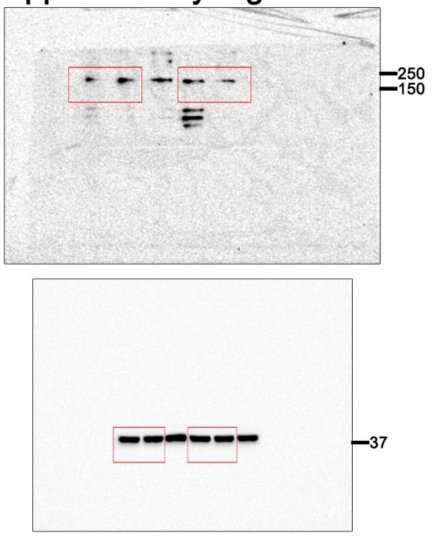

Supplementary Figure 2d

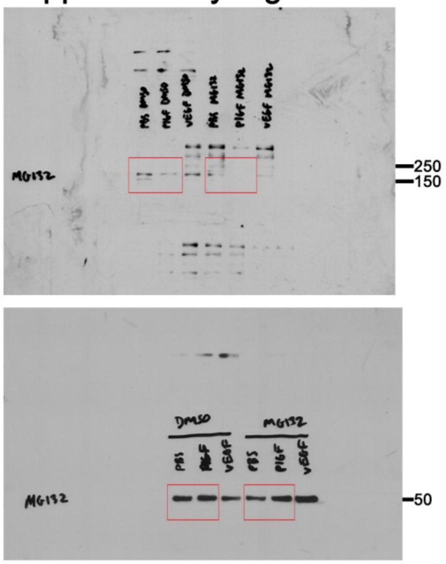

Uncropped blots for Supplementary Figure 4

Supplementary Figure 4b

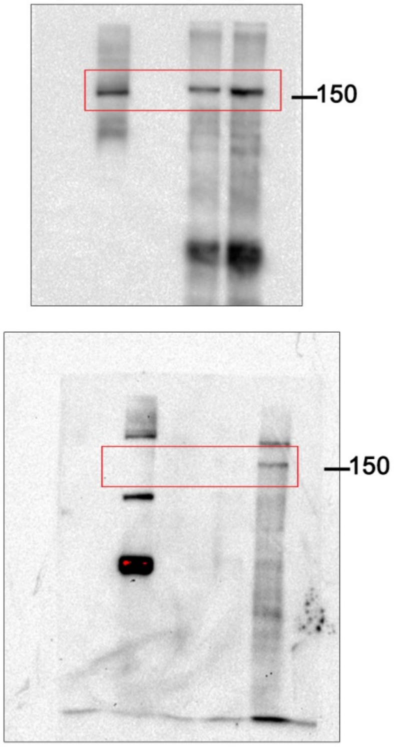

Supplementary Figure 4e

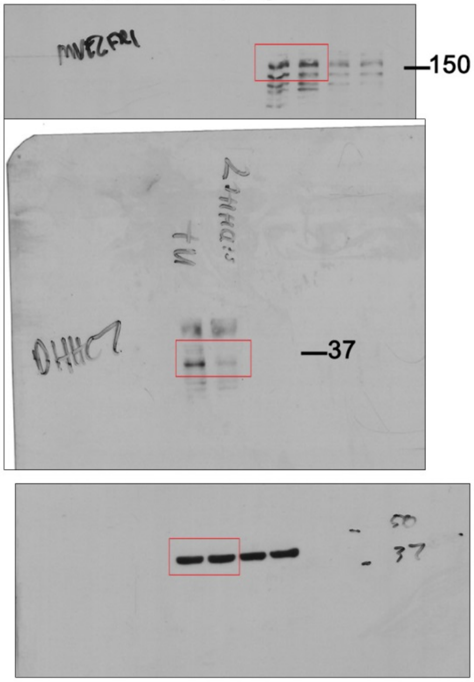

Supplementary Figure 4d

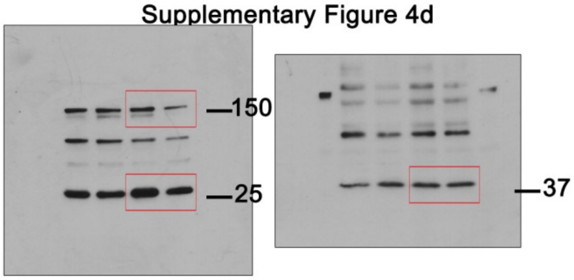

Uncropped Blots for Supplementary Figure 5

Supplementary Figure 5c

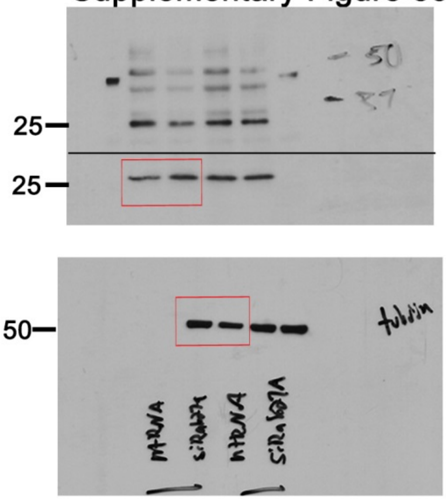

Supplementary Figure 5d

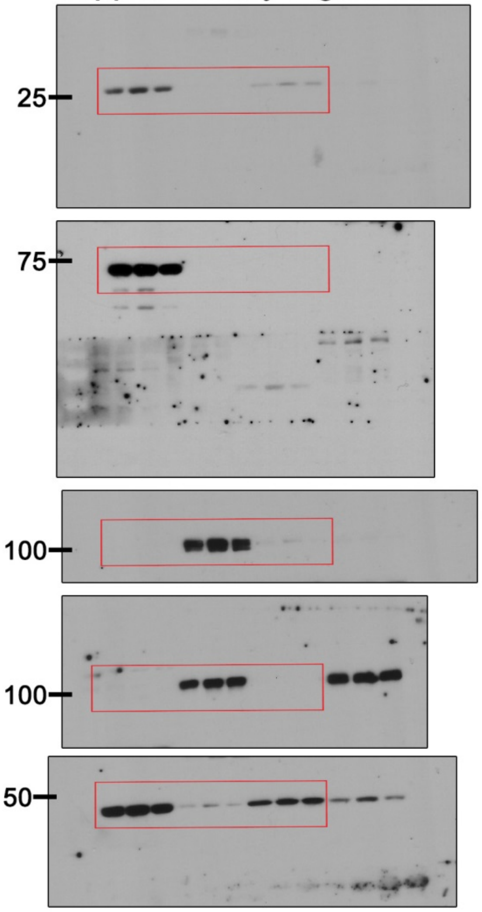

Uncropped Blots for Supplementary Figure 6

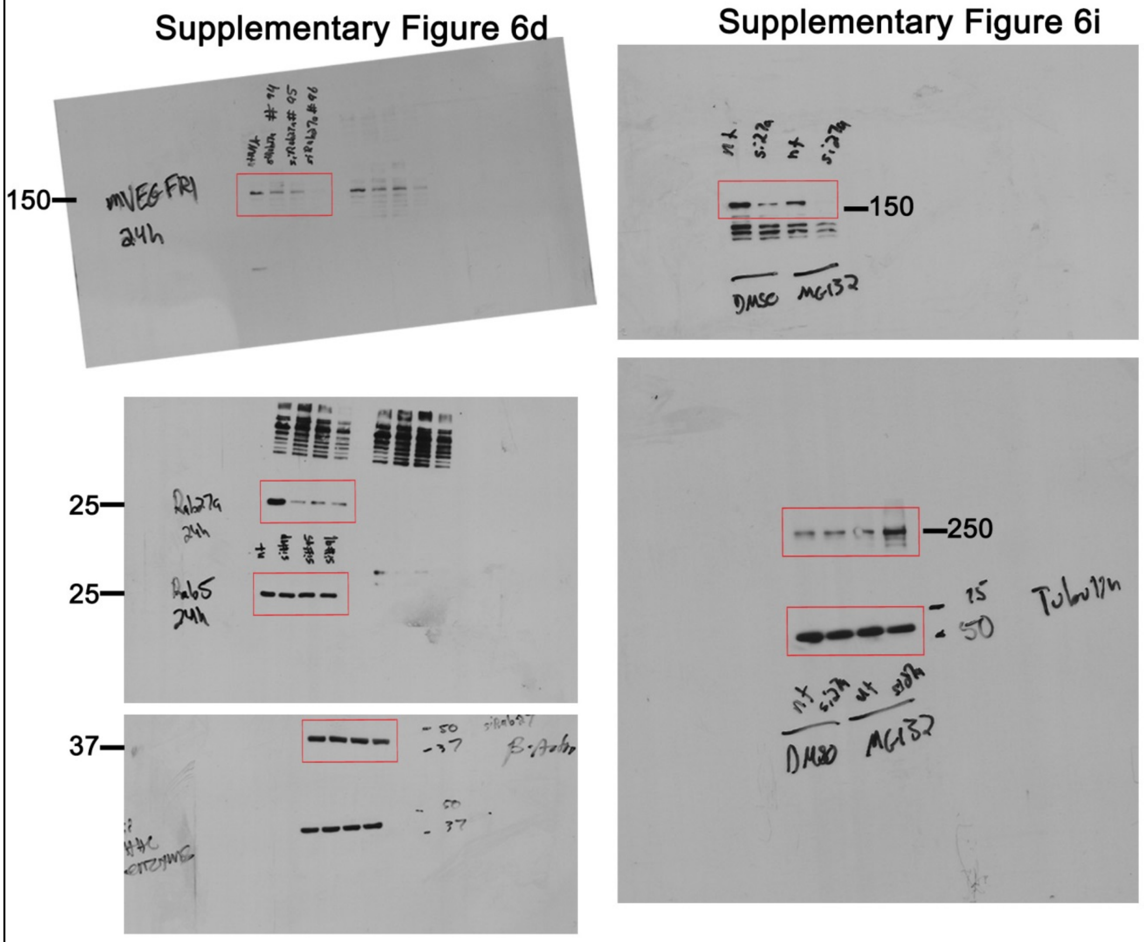

Uncropped Blots for Supplementary Figure 7

Supplementary Figure 7e

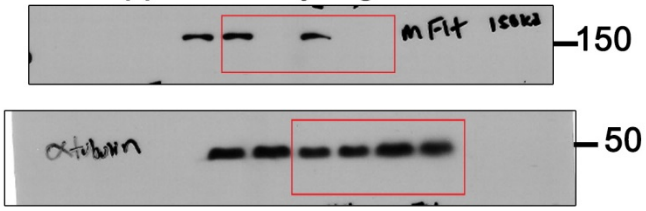

Supplemental Figure 7f

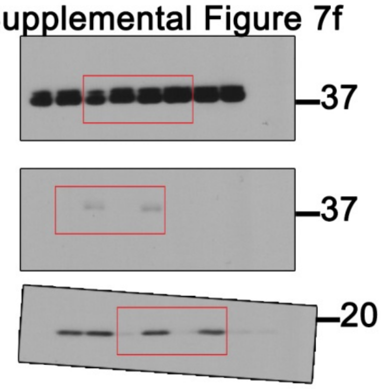

Uncropped Blots for Supplementary Figure 8

Supplementary Figure 8b

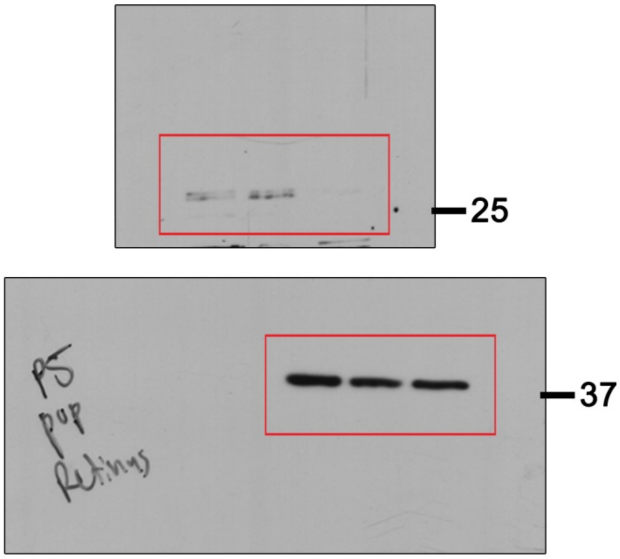

Supplementary Table 1. List of antibodies used for this study.

Antibody	Company	IB Titer	IF Titer
**PRIMARY**			
α-tubulin	Sigma	1:10000	-
β-actin	CST	1:10000	-
DHHC3	Abcam	1:4000	-
DHHC3	SCBT	1:2000	1:750
Erk1/2 (4695)	CST	1:5000	-
p-Erk1/2 (4370)	CST	1:1000	1:500
GM130 (ab-52649)	Abcam	1:2000	-
HA	CST	1:5000	-
P-Histone H3 (9701)	CST	1:1000	1:1000
LAMP1	Abcam	-	1:2000
LYPLA1 (APT1)	Abcam	1:2000	-
PKM1/2 (3186S)	CST	1:1000	-
P-VEGFR2 Y1175 (2478)	CST	1:500	-
PECAM	Abcam	-	1:500
Rab3a (MN1250)	Thermo Scientific	1:750	-
Rab4/14 (sc-376243)	SCBT	1:1000	-
Rab5	SCBT	1:500	-
Rab11 (sc-166912)	SCBT	1:2000	1:750
Rab27a (sc-81914)	SCBT	1:1000	1:500
Streptavidin HRP (N100)	Life Technologies	1:5000	-
TGN46	Abcam	-	1:2000
VE-Cadherin (sc-28644)	SCBT	1:1000	-
VEGFR1 (ab-32152)	Abcam	1:2000	1:500*
Alexa-fluor 488-VEGFR1 (ab-195253)	Abcam	-	1:50
VEGFR2 (2479)	CST	1:5000	1:1000
ZDHHC21 (ab-103755)	Abcam	1:750	-
**SECONDARY**			
Anti-mouse IgG, HRP-linked (secondary)	CST	1:5000	-
Anti-rabbit IgG, HRP-linked (secondary)	CST	1:5000	-
Goat anti-mouse 488	Life Technologies	-	1:1000
Goat anti-mouse 594	Life Technologies	-	1:1000
Goat anti-rabbit 488	Life Technologies	-	1:1000
Goat anti-rabbit 594	Life Technologies	-	1:1000

*A titer of 1:200 was used for Flt-1 staining in the retina

Abbreviations:

CST= Cell Signaling Technologies

SCBT= Santa Cruz Biotechnology

Supplementary Table 2. List of Inhibitors used for this study.

Inhibitors	Concentration	Company	Target
2-Bromohexadecanoic Acid (2-BH)	20 µM in EtOH	Sigma	Palmitoyl-acetyl transferases
Chloroquine (CHQ)	10 µg/mL in H2O	Sigma	Lysosome
Cycloheximide (CHX)	500 µg/mL in H2O	Sigma	Ribosome
Dynasore	1µM in DMSO	Santa Cruz	Dynamin
MG132	5µM in DMSO	Calbiochem	Proteasome
Palmostatin B (Pal-B)	10 µM in DMSO	Calbiochem	Acyl-protein thioesterases

Supplementary Table 3. List of small interfering RNA knockdown probes used for this study.

siRNAs	Catalogue #	Company	Pmol/10^6 cells	Target species
APT1	S20410	ThermoFisher	100	Human
DHHC3	S27899	ThermoFisher	125	Human
DHHC3 #2	S27900	ThermoFisher	125	Human
DHHC7	S31109	ThermoFisher	125	Human
Flt-1	SI00031465	Qiagen	75	Human
Non-targeting	SI03650318	Qiagen	varies	none
Rab4a	S11677	ThermoFisher	100	Human
Rab11a	S16702	ThermoFisher	100	Human
Rab27a#1*	SI02662744	Qiagen	100	Human
Rab27a#2**	S11695	ThermoFisher	-	Human/mouse
Rab27a # 3	S11494	ThermoFisher	100	Human/mouse

*Used for all human cell work in 2D and 3D.

**Used for mouse aortic ring assay (200pmol) and to validate effects of Rab27a-1 probe. Used at 100pmol/10^6 cell for Rab27a knockdown validation in Supplementary Figure 6d.
